# Phylogenetic Reassessment of Antarctic Tetillidae (Demospongiae, Tetractinellida) Reveals New Genera and Genetic Similarity among Morphologically Distinct Species

**DOI:** 10.1371/journal.pone.0160718

**Published:** 2016-08-24

**Authors:** Mirco Carella, Gemma Agell, Paco Cárdenas, Maria J. Uriz

**Affiliations:** 1 Centre d’Estudis Avançats de Blanes (CEAB-CSIC), Accés Cala St Francesc 14, 17300 Blanes (Girona), Spain; 2 Département Milieux et Peuplements Aquatiques, Muséum National d’Histoire Naturelle, UMR 7208 “BOrEA”, Paris, France; 3 Department of Medicinal Chemistry, Division of Pharmacognosy, BioMedical Centre, Husargatan 3, Uppsala University, 751 23 Uppsala, Sweden; Tierarztliche Hochschule Hannover, GERMANY

## Abstract

Species of Tetillidae are distributed worldwide. However, some genera are unresolved and only a few genera and species of this family have been described from the Antarctic. The incorporation of 25 new COI and 18S sequences of Antarctic Tetillidae to those used recently for assessing the genera phylogeny, has allowed us to improve the resolution of some poorly resolved nodes and to confirm the monophyly of previously identified clades. Classical genera such as *Craniella* recovered their traditional diagnosis by moving the Antarctic *Tetilla* from *Craniella*, where they were placed in the previous family phylogeny, to *Antarctotetilla* gen. nov. The morphological re-examination of specimens used in the previous phylogeny and their comparison to the type material revealed misidentifications. The proposed monotypic new genus *Levantinella* had uncertain phylogenetic relationships depending on the gene partition used. Two more clades would require the inclusion of additional species to be formally established as new genera. The parsimony tree based on morphological characters and the secondary structure of the 18S (V4 region) almost completely matched the COI M1-M6 and the COI+18S concatenated phylogenies. Morphological synapomorphies have been identified for the genera proposed. New 15 28S (D3-D5) and 11 COI I3-M11 partitions were exclusively sequenced for the Antarctic species subset. Remarkably, species within the Antarctic genera *Cinachyra* (*C*. *barbata* and *C*. *antarctica*) and *Antarctotetilla* (*A*. *leptoderma*, *A*. *grandis*, *and A*. *sagitta*), which are clearly distinguishable morphologically, were not genetically differentiated with any of the markers assayed. Thus, as it has been reported for other Antarctic sponges, both the mitochondrial and nuclear partitions used did not differentiate species that were well characterized morphologically. Antarctic Tetillidae offers a rare example of genetically cryptic (with the traditional markers used for sponges), morphologically distinct species.

## Introduction

Sponges dominate some benthic communities in the Antarctic, in terms of both biomass [1], [2] and diversity [3]. The Antarctic clockwise circumpolar current [4] and the low water temperatures contribute to the biogeographic isolation of the Antarctic continental shelf, which partly explains the high degree of sponge endemism in the area [5], [6], [7]. Taxonomic affinities between Antarctic sponges and those of the Magellanic region (South America) and the Falkland Islands have also been reported [8], [5], [9] but the studies are still incomplete and subject to debate [10], [7]. Most of the currently known Antarctic sponge species were discovered during the oceanographic campaigns of the twentieth century [11], [12], [13], [14], [8], [15], [16], [17], [18], [19], [20], [21], [22], [23]. However, recent findings of many new species [24], [7], [25], [26], [27], [28], [29], [30], [31], [32] suggest that the sponge biodiversity of this area has not been fully explored yet and that more species remain to be discovered.

While collecting sponges during the Antarctic Polarstern ANT-XXVII/3 expedition in 2011, we realized the difficulty to identify the fairly common, well known, large conspicuous species belonging to the family Tetillidae Sollas, 1886. The World Porifera Database [33] currently lists only four valid Tetillidae species from the Antarctic—*Cinachyra barbata* (Sollas, 1886), *Cinachyra antarctica* (Carter, 1872), *Tetilla leptoderma* (Sollas, 1886) and *Craniella sagitta* (Lendenfeld, 1907). Furthermore, intra-specific variations of the mitochondrial cytochrome c oxidase subunit 1 (COI), from Antarctic and New Zealand Tetillidae, also suggest that the diversity of this group is underestimated [34]. We also noticed that there is no consensus in the literature regarding the allocation of some Antarctic species to the genus *Craniella* or *Tetilla* [35], [36], [34].

The family Tetillidae (Demospongiae, Tetractinellida, Spirophorina) contains 156 species distributed worldwide [33]. Many of them inhabit sedimentary bottoms to which they anchor by means of long spicule bundles, which represent a suitable substrate for many other hard-bottom invertebrates [37]. Their representatives are characterized by a globular habit, a radiate skeleton composed chiefly of the following spicules: megascleres are protriaenes, oxeas, and sometimes ortho/anatriaenes or calthrops, which often protrude the ectosomal layer outward; microscleres are characteristic sigmaspires and occasionally raphides [36]. To this day, the Tetillidae have no clear morphological synapomorphy, as triaenes are shared with all Tetractinellida, and sigmaspires are found in most Spirophorina families. The Tetillidae appears monophyletic with COI [38], but polyphyletic in 18S and 28S (C1-D2) phylogenies [39], [40], [38].

Using the COI of 14 Tetillidae species, Szitenberg et al. [41] suggested that most Tetillidae genera were not monophyletic. Later, Szitenberg et al. [34], using this time a set of three molecular markers (COI, 28S and 18S) on 28 Tetillidae species belonging to eight genera, obtained five main clades (with COI and 28S): (i) *Acanthotetilla* (ii) *Cinachyrella*, *Paratetilla*, and *Amphitethya*, (iii) *Cinachyrella levantinensis*, (iv) tropical-temperate *Tetilla*, and (v) *Craniella*, *Cinachyra*, and *Fangophilina*. Results were similar with 18S, except that *Acanthotetilla* sequences were lacking from the NCBI genbank. One of the main issues raised by this study concerned the *Craniella/Cinachyra/Fangophilina* clade, which included all the Antarctic species. Results suggested the polyphyly of the genus *Craniella* distributed in three clades: (i) *Craniella* cf. *leptoderma* (Antarctic, New Zealand), (ii) *Craniella sagitta* (Antarctic, New Zealand) and (iii) a *Craniella* clade with boreo-arctic Atlantic species mixed with New Zealand/Australian species. Based on this polyphyly, Szitenberg et al. [34] propose to reallocate the Antarctic *Tetilla*, *Fangophilina*, and *Cinachyra* to the genus *Craniella*, despite the absence of morphological support for such a proposal.

The goal of the current study was to investigate the relationships between the Antarctic and tropical/temperate Tetillidae to revise the taxonomy and phylogeny of the family and to assess the purported endemism of its Antarctic genera. We improved the sampling of previous phylogenies by incorporating additional Antarctic and Sub-Antarctic specimens from geographically distant localities. We used four gene partitions (mitochondrial and nuclear) to conduct molecular phylogenetic analyses. The morphology of new specimens, type species, and some specimens previously sequenced [34] was examined. Finally, we also performed a maximum parsimony phylogenetic analysis based on morphological characters and the secondary structure of the V4 region of the 18S rDNA.

Our findings confirm the monophyly of the Antarctic genera and allow us to erect two new Tetillidae genera: *Antarctotetilla* gen. nov., restricted to Antarctic and sub-Antarctic waters, and *Levantinella* gen. nov. so far limited to eastern Mediterranean. This study also resurrects a sub-Antarctic species and reveals four potential new species. The genetic homogeneity of the markers used among morphologically distinct Antarctic Tetillidae species, contrasts with the habitual finding of genetically distinct, morphologically cryptic species. A restricted geographical distribution of some Tetillidae genera has become evident.

## Material and Methods

The collection of sponge samples was conducted in strict accordance with Spanish and European regulations under the rules of the Spanish National Research Council with the approval of the Directorate of Research of the Spanish Government. The study was found exempt from ethics approval by the ethics commission of the University of Barcelona since, according to article 3.1 of the European Union directive (2010/63/UE) from the 22/9/2010, no approval is needed for sponge sacrification, as they are the most primitive Animals and lack any nervous system Moreover, the collected sponges are not listed in CITES."

### Sampled sponges

The majority of the samples were collected in Antarctic and sub-Antarctic regions during the Polarstern ANT-XXVII/3 expedition from Punta Arenas, Chile (February 8, 2011) to Cape Town, South Africa (18 April 2011) with Agassiz (AGT) and bottom trawl (BT) gears. During this expedition, Tetillidae were collected in South Georgia, South Orkneys Islands, and Newmayer in the Antarctic continent. A Remote Operated Vehicle (ROV) was deployed during the Polarstern cruise to gather samples between 300 and 450 meters and to photograph underwater living specimens. Given the large size (up to 30 cm in diameter) of most Antarctic Tetillidae, once the individuals were photographed, a fragment ca. 3 cm^3^ in size was preserved in absolute ethanol, which was changed three times before packing at -20°C for transportation and storage at the CEAB (Centre d’Estudis Avançats de Blanes, Spain). Other Tetillidae from this study were collected during the Collaborative East Antarctic Marine Census (CEAMARC) Dec. 2007- Jan. 2008 in Adélie Land, Antarctica [42], [43]. The CEAMARC specimens were dredged between 170 and 1700 m; the complete specimens were bulk-fixed with ethanol (80%) in 60L metallic drums. A few samples also came from a fishery-independent biomass survey “POissons de KERguelen” (POKER II) conducted in 2010 on the Kerguelen Plateau. The complete specimens were collected using a Grande Ouverture Verticale (GOV) trawl, frozen on board and bulk-fixed in ethanol 80% at the Muséum National d’Histoire Naturelle (MNHN), Paris, France. Specimens from these two expeditions are housed at the MNHN and stored in the ‘Zoothèque’ at a constant 18°C temperature. Three additional samples were collected between Lavoisier and the Antarctic Peninsula between 847 and 960 m (R/V LM Gould, 2010) and were obtained from Bill Baker (University of South Florida). The samples used in this study, voucher numbers, Genbank accession numbers, and collecting localities are provided in Table 1.

**Table 1:** List of species used in the study, with collection reference number, accession number of the sequences stored in the Genbank, revised species name, and geographical origin.

		Genbank accession numbers					
SPECIES	Voucher number	COI M1-M6	18S	COI I3-M11	28S (D3-D5)	Revised species name	Collection sites
*Tetilla leptoderma*	ANT 27111	**KT124318**	**KT124341**	**KT124328**	**KT124362**	*Antarctotetilla leptoderma*	Sub Antarctic (South Georgia)
*Tetilla leptoderma*	ANT 27112	**KT124319**	**KT124343**	**KT124329**	**KT124365**	*Antarctotetilla leptoderma*	Antarctica (Newmayer)
*Tetilla grandis*	ANT 27123	**KT124324**	**KT124344**	**KT124330**	**KT124363**	*Antarctotetilla grandis*	Antarctica (Newmayer)
*Tetilla grandis*	ANT 27124	**KT124325**	**KT124346**	**KT124331**	**KT124364**	*Antarctotetilla grandis*	Antarctica (Newmayer)
*Cinachyra barbata*	ANT 27212	**KT124314**	**KT124356**	**KT124336**	**_**		Sub Antarctic (South Georgia)
Tetillidae	ANT 27211	**KT124313**	**KT124355**	**_**	**KT124361**	Tetillidae sp. 3	Sub Antarctic (South Orkneys)
*Cinachyra antarctica*	ANT 27204	**KT124317**	**_**	**_**	**KT124367**		Antarctica (Newmayer)
*Tetilla leptoderma*	ANT 27107	**_**	**KT124351**	**_**	**KT124358**	*Antarctotetilla leptoderma*	Antarctica (Newmayer)
*Tetilla leptoderma*	ANT 27108	**KT124323**	**KT124347**	**_**	**_**	*Antarctotetilla leptoderma*	Sub Antarctic (Souh Orkneys)
*Tetilla leptoderma*	ANT 27109	**_**	**KT124354**	**_**	**_**	*Antarctotetilla leptoderma*	Antarctica (Newmayer)
*Cinachyra antartica*	ANT 27223	**_**	**KT124353**	**KT124335**	**KT124368**		Antarctica (Newmayer)
*Cinachyra barbata*	ANT 27205	**KT124321**	**KT124340**	**_**	**KT124366**		Antarctica (Newmayer)
*Tetilla leptoderma*	ANT 27105	**KT124322**	**KT124348**	**_**	**KT124359**	*Antarctotetilla leptoderma*	Antarctica (Newmayer)
*Tetilla leptoderma*	ANT 27106	**_**	**KT124349**	**_**	**KT124360**	*Antarctotetilla leptoderma*	Antarctica (Newmayer)
*Tetilla grandis*	MNHN-Poker II-Chalut 32 sp.4	**KT124326**	**KT124345**	**_**	**_**	*Antarctotetilla grandis*	Sub Antarctic (Kerguelen)
*Cinachyra antarctica*	MC 7485	**KT124316**	**KT124339**	**_**	**_**		Between Lavoisier and Antarctica
*CInachyra antarctica*	MC 7486	**KT124315**	**_**	**_**	**_**		Between Lavoisier and Antarctica
*Tetilla sagitta*	MNHN-IP 2009 359	**KT124327**	**_**	**KT124334**	**_**	*Antarctotetilla sagitta*	Antarctica (Adelie Land)
*Cinachyra barbata*	MNHN-IP 2009 506a	**KT124312**	**KT124350**	**_**	**_**		Antarctica (Adelie Land)
*Tetilla sagitta*	MNHN-IP 2009 351	**_**	**KT124352**	**KT124333**	**KT124369**	*Antarctotetilla sagitta*	Antarctica (Adelie Land)
*Cinachyra barbata*	MNHN-IP 2009 387	**_**	**KT124342**	**_**	**_**		Antarctica (Adelie Land)
*Tetilla sagitta*	MNHN-IP 2009 31	**KT124320**	**_**	**KT124332**	**KT124370**	*Antarctotetilla sagitta*	Antarctica (Adelie Land)
*Cinachyra barbata*	NIWA 28877	JX177864	JX177977	**_**	**_**	*Cinachyra* cf. *barbata*	Antarctica (Oates land)
*Cinachyra antarctica*	NIWA 28951	JX177868	**_**	**_**	**_**		Antarctica (Oates land)
*Cinachyra antarctica*	NIWA 28957	JX177867	**_**	**_**	**_**		Antarctica (Oates land)
*Cinachyra antarctica*	QMG 311149	JX177914	**_**	**_**	**_**	*Cinachyra* sp.	Antarctica, Ross island (Mcmurdo base)
*Craniella sagitta*	NIWA 25206	JX177917	JX177981	**_**	**_**	Tetillidae sp.2	New Zealand (Chatham rise)
*Craniella sagitta*	NIWA 28491	JX177915	**_**	**_**	**_**	Tetillidae sp.2	New Zealand (Chatham rise)
*Craniella sagitta*	NIWA 28929	JX177863	**_**	**_**	**_**	Tetillidae sp.1	Antarctica (Oates land)
*Craniella* cf. *leptoderma*	NIWA 28910	JX177865	JX177982	**_**	**_**	*Antarctotetilla* cf. *grandis*	Antarctica (Oates land)
*Craniella* cf. *leptoderma*	NIWA 36097	JX177866	**_**	**_**	**_**	*Antarctotetilla grandis*	Antarctica (Ross island)
*Craniella* cf. *leptoderma*	NIWA 52077	JX177916	**_**	**_**	**_**	*Antarctotetilla leptoderma*	New Zealand (Chatham rise)
*Craniella* cf. *leptoderma*	NIWA 28496	JX177897	**_**	**_**	**_**	*Antarctotetilla leptoderma*	New Zealand (Chatham rise)
*Craniella* cf. *leptoderma*	NIWA 28524	JX177895	JX177976	**_**	**_**	*Antarctotetilla leptoderma*	New Zealand (Chatham rise)
*Craniella* cf. *leptoderma*	NIWA 28507	JX177896	JX177975	**_**	**_**	*Antarctotetilla leptoderma*	New Zealand (Chatham rise)
*Craniella* sp.	QMG 316342	HM032747	JX177983	**KT124337**	**KT124371**	*Cinachyra* sp.	Australia (South Norfolk ridge)
*Craniella zetlandica*	PC 252	KC122679	**_**	**_**	**_**		Røst reef, Norway
*Craniella zetlandica*	VM 14754	**_**	JX177986	**_**	**_**		Iceland
*Craniella neocaledoniae*	NIWA 28591	**_**	JX177984	**_**	**_**		New Zealand
*Craniella* sp.	QMG 318785	HM032752	JX177985	**_**	**_**		Australia (South Norfolk ridge)
*Craniella* sp.	BIOICE 3659	HM032750	**_**	**_**	**_**		Iceland
*Craniella* sp.	QMG 316372	HM032748	HE591469	**KT124338**	**KT124372**	*Cinachyra* sp.	Australia (South Norfolk ridge)
*Craniella* sp.	ZMBN 85240	HM592668	**_**	**_**	**_**	*Craniella* cf. *cranium*	Norway
*Craniella cranium*	ZMBN 85239	HM592669	**_**	**_**	**_**	*Craniella* aff. *zetlandica*	Norway
*Fangophilina* sp.	NIWA 28601	JX177919	JX177979	**_**	**_**	cf. *Fangophillina*	New Zealand (Challenger Plateau)
*Fangophilina* sp.	NIWA 28586	JX177918	JX177978	**_**	**_**	cf. *Fangophillina*	New Zealand (Challenger Plateau)
*Fangophilina* sp.	NIWA 28617	JX177912	JX177980	**_**	**_**	cf. *Fangophillina*	New Zealand (Challenger Plateau)
*Paratetilla* sp.	QMG 314224	HM032744	**_**	**_**	**_**		Australia (Curacoa Island)
*Cinachyrella schulzei*	QMG 320143	HM032746	**_**	**_**	**_**	*Cinachyrella* cf. *tenuiviolacea*	Australia (Keppel Islands)
*Cinachyrella schulzei*	QMG 320636	HM032745	JX177971	**_**	**_**	*Cinachyrella* cf. *tenuiviolacea*	Australia (Melanie Patches)
*Cinachyrella apion*		AJ843895	**_**	**_**	**_**		Bermuda
*Cinachyrella* sp.	TAU 25622	**_**	JX177962	**_**	**_**		Tanzania
*Cinachyrella* sp.	TAU 25621	HM032740	JX177964	**_**	**_**		Tanzania
*Cinachyrella* sp.	QMG 320270	HM032741	JX177963	**_**	**_**		Australia (Wellington point, Moreton Bay)
*Craniella* cf. *leptoderma*	QMG 315031	HM032749	JX177974	**_**	**_**	*Antarctotetilla* cf. *sagitta*	Antartica (Casey Antartic Research Base)
*Cinachyrella australiensis*	QMG 321405	HM032743	**_**	**_**	**_**		Australia (Sunshine Coast)
*Cinachyrella australiensis*	QMG 320216	JX177902	JX177966	**_**	**_**		Australia (Keppel Islands)
*Cinachyrella australiensis*	QMG 320656	**_**	JX177968	**_**	**_**		Australia (Munro Reef, Coral Sea)
*Cinachyrella australiensis*	QMG 320656	**_**	JX177967	**_**	**_**		Australia
*Cinachyrella apion*	ZMBN 81789	HM592667	**_**	**_**	**_**		USA
*Cinachyrella apion*	SBP-B25	EF519601	**_**	**_**	**_**		Caribean Sea
*Cinachyrella apion*		FJ711645	**_**	**_**	**_**		Panama
*Cinachyrella apion*		**_**	AJ627186	**_**	**_**		Bermuda
*Cinachyrella* cf. *paterifera*	0M9H2022-P	**_**	KC902343	**_**	**_**		Australia
*Cinachyrella* sp.	USNM 1204826	**_**	KC901899	**_**	**_**		Panama
*Cinachyrella* sp.	USNM 1204829	**_**	KC902189	**_**	**_**		Panama (Bocas del Toro)
*Cinachyrella alloclada*	DH S271	JX177913	JX177965	**_**	**_**		Panama
*Cinachyrella alloclada*	USNM 1133831	**_**	KC902108	**_**	**_**		Panama
*Cinachyrella alloclada*	0M9G1250-W	**_**	KC902264	**_**	**_**		USA
*Cinachyrella alloclada*	TAU 25623	HM032738	**_**	**_**	**_**		Bahamas
*Tetilla radiata*	MNRJ 576	HM032742	**_**	**_**	**_**		Brazil (Rio De janeiro)
*Tetilla murycii*	UFBA 2586POR	JX177898	**_**	**_**	**_**		Brazil (Camamu Bay)
*Cinachyrella levantinensis*	TAU 25529	JX177906	JX177970	**_**	**_**		Lebanese Coast
*Cinachyrella levantinensis*	TAU 25568	JX177904	JX177969	**_**	**_**	* Levantinella levantinensis*	Lebanese Coasts
*Cinachyrella levantinensis*	MHNM 16194	JX177905	HM629803	**_**	**_**	* Levantinella levantinensis*	Lebanese Coast
*Cinachyrella levantinensis*	DH S124	JX177903	**_**	**_**	**_**	*Levantinella levantinensis*	Lebanese Coast
*Cinachyrella levantinensis*	TAU 25456	**_**	HM629802	**_**	**_**	*Levantinella levantinensis*	Lebanese Coast
*Tetilla japonica*		**_**	TTL18SR	**_**	**_**		Japan
*Tetilla japonica*	TAU 25619	JX177901	**_**	**_**	**_**		Japan
*Cinachyrella* sp.	SP.11	**_**	AY734439	**_**	**_**		Australia?
*Cinachyrella* sp.	SP.22	**_**	AY734437	**_**	**_**		Australia?
*Cinachyrella* sp.	SP.24	**_**	AY734438	**_**	**_**		Australia?
*Cinachyrella kuekenthali*	SBP-K75	EF519603	**_**	**_**	**_**		Caribean Sea
*Cinachyrella kuekenthali*	SBP-B79	EF519602	**_**	**_**	**_**		Caribean Sea
*Cinachyrella kuekenthali*		FJ711646	**_**	**_**	**_**		Panama
*Cinachyrella kuekenthali*		NC010198	**_**	**_**	**_**		USA
*Cinachyrella kuekenthali*		EU237479	**_**	**_**	**_**		USA
*Paratetilla bacca*	TAU 25620	JX177900	**_**	**_**	**_**		Thailand
*Paratetilla bacca*	LB 622	JX177894	**_**	**_**	**_**		Indonesia
*Paratetilla bacca*	LB 671	JX177893	JX177972	**_**	**_**		Indonesia
*Paratetilla bacca*	0M9H2290-H	**_**	KC902195	**_**	**_**		Australia
*Amphitethya* cf. *microsigma*	SAM S1189	JX177910	**_**	**_**	**_**	*Amphitethya microsigma*	South Australia?
*Acanthotetilla celebensis*	RMNH POR 2877	JX177893	**_**	**_**	**_**		Indonesia
*Acanthotetilla walteri*	UFBA 2021	JX177907	**_**	**_**	**_**		Brazil
*Acanthotetilla seychellensis*	0CDN 8107-V	**_**	KC902033	**_**	**_**		American Samoa
*Cinachyrella kuekenthali*	USNM 1133786	**_**	KC902290	**_**	**_**		Panama
*Cinachyrella kuekenthali*		**_**	EU702414	**_**	**_**		USA
*Cinachyra* sp.	0CDN 8726-T	**_**	KC902124	**_**	**_**		Guam
*Craniella* sp.	0CDN 5142-X	**_**	KC902265	**_**	**_**		Philippines
*Geodia cydonium*		**_**	AY348878	**_**	**_**		Mediterranean Sea
*Geodia cydonium*	ZMA POR 21652	HM592738	**_**	**_**	**_**		Portugal
*Geodia neptuni*		**_**	AY737635	**_**	**_**		Caribean Sea
*Thenea levis*	ZMBN 85230	HM592717	**_**	**_**	**_**		Norway
*Theonella swinhoei*	ZMA POR 16637	HM592745	**_**	**_**	**_**		Egypt

Reference numbers of individuals sequenced *de novo* in the current study are indicated in bold. Abbreviations: BIOICE, The inter-Nordic BIO-Iceland project; DH, LB, personal collections of Dorothée Huchon and Lisa Becking; MC, National Museums, Northern Ireland, Holywood; MHNM, Muséum d’Histoire Naturelle Palais Longchamp, Marseille, France;MNHM, Muséum National d’Histoire Naturelle, Paris, France; MNRJ–Museu Nacional do Rio de Janeiro, Brazil; NIWA, National Institute of Water & Atmospheric Research, New Zealand; PC, personal collection, University of Bergen, Norway; QMG, Queensland Museum, Australia; RMNH, Rijksmuseum van Natuurlijke Historie, Leiden, Nederland; SAM, South Australian Museum, Australia; SBP, Sponge Barcoding Project (http://www.palaeontologie.geo.uni-muenchen.de/SBP/); TAU, Steinhardt National Collection of Natural History, Zoological Museum at Tel Aviv University, Israel; UFBA, Universidade Federal da Bahia, Brazil; USNM, United States National Museum, U.S.A.; VM, Museum of Natural History and Archaeology, a part of the University of Science and Technology, Trondheim, Norway; ZMA, Zoölogisch Museum van de Universiteit van Amsterdam, Holland; ZMBN, Zoologisk Museum, Bergen, Norway; 0CDN, 0M9G, Smithsonian Institution/National Museum of Natural History, U.S.A.

Additional specimens of particular interest to obtain a more comprehensible sampling for our taxonomic study and to verify previous identifications were obtained on loan from several institutions: paratype of *Fangophilina submersa* Schmidt, 1880 (MSZ.PO160, Musée Zoologique de Strasbourg, France); paratype of *Craniella quirimure* Peixinho, Cosme, Hajdu, 2005 (MNRJ 8417, Museu nacional/UFRG, Brazil); paratype of *Tetilla radiata* Selenka, 1879 (MNRJ 576, Museu nacional/UFRG, Brazil); *Tetilla muricyi* Fernandez, Peixinho, Pinheiro, Menegola, 2011 (UFBA 2569, Museu de História Natural da Bahia, Brazil); *Craniella* sp. (QMG 316342, and QMG 316372, Queensland Museum, Brisbane, Australia); nine individuals of *Cinachyrella levantinensis* Vacelet, Bitar, Carteron, Zibrowius, Pérez, 2007 from Lebanon (06/07/2003-1 and 31/07/2003-2, Station Marine d’Endoume, Marseille) and seven specimens collected across the shore of Ma’agan Michael, Israel (courtesy of Jean Vacelet); *Craniella sagitta* Lendenfeld, 1907 (syn. *Tethya sagitta*) (NIWA 28491 and NIWA 28929 National Institute of Water & Atmospheric Research, New Zealand); a small piece of the syntype of *Tethya sagitta* Lendenfeld, 1907 (ZMB Por 3504, Museum für Naturkunde Leibniz, Germany); *Fangophilina* sp. (NIWA 28601 National Institute of Water & Atmospheric Research, New Zealand). Moreover, several specimens from Szitenberg et al. [34] were re-examined from photographs or specimens (see Results).

Selected outgroups for the phylogenetic analyses, which mainly aimed at establishing relationships among genera, belonged to the Astrophorina (families Geodiidae and Theneidae) since previous molecular phylogenies of Demospongiae based on mitochondrial [44], [38] and nuclear [45], [46] genes placed Astrophorina either as a sister clade of the Tetillidae (COI), or sister to some Tetillidae (18S, 28S).

### DNA extraction, amplification and sequencing

Genomic DNA was extracted according to the manufacturer’s protocol for the DNeasy Blood & Tissue kit (Qiagen). Two mitochondrial markers were sequenced, both from COI: the M1-M6 partition, using primers LCO1490 and HCO2198 [47] and the I3-M11 partition, using primers PorCOI2 fwd. and PorCOI2 rev. [48]. Two nuclear markers were also sequenced: 18S, using primers 1F and 1795R, [49] and the D3-D5 partition of 28S, using primers Por28S-830F and Por28S-1520R [50]. Different amplification protocols were performed for each marker: COI M1-M6 partition (94°C, 2 min [94°C, 1 min, 43°C, 1 min, 72°C, 1 min] x 35–40 cycles, 72°C, 5 min); COI I3-M11 partition (95°C, 3 min, [94°C, 30 s, 57°C, 45 s, 72°C, 90 s] x 35–40 cycles, 72°C, 10 min); 18S (94°C, 5 min, [94°C, 1 min, 50–55°C, 1 min, 72°C, 1 min] x35-40 cycles, 72°C, 5 min); 28S D3-D5 partition (94°C, 5 min [94°C,30 s, 53°C, 30 s, 72°C,30 s] x 30 cycles, 72°C, 5 min). COI M1-M6 partition amplifications were performed in a 50 μL volume reaction, containing 37,6 μL H₂0, 5 μL buffer KCL (BIORON), 2μL BSA, 2μL dNTP (Sigma), 1 μL primers forward/reverse, 0,4 μL Taq (BIORON) and 1μL of genomic DNA. Amplifications of the COI I3-M11 partition were performed in a 50 μL volume reaction, containing 34,45 μL H₂0, 5 μL buffer (INVITROGEN), 0,75 μL MgCl (INVITROGEN), 2,4 μL DMSO (dimethyl sulfoxide), 2 μL BSA, 2 dNTP (Sigma), 1 μL primers forward/reverse, 0,4 Taq (INVITROGEN) and 1 μL of genomic DNA. Amplifications of 18S rRNA were performed in a 50 μL volume reaction, containing 36,85 μL H₂0, 5 μL buffer (INVITROGEN), 0,75 μL MgCl (INVITROGEN), 1,2 μL DMSO (dimethyl sulfoxide), 1 μL BSA, 1,5 μL dNTP (Sigma), 1 μL primers forward/reverse, 0,7 μL Taq (INVITROGEN) and 1 μL of genomic DNA. On the other hand, partition D3-D5 of 28S rRNA amplifications were performed in a 50 μL volume reaction, containing 36,85 μL H₂0, 5 μL buffer (INVITROGEN), 0,75 μL MgCl (INVITROGEN), 2μL BSA, 2 μL dNTP (Sigma), 1 μL primers forward/reverse, 0,4 μL Taq (INVITROGEN) and 1 μL of genomic DNA. Purified PCR products were sequenced in both directions using Applied Biosystems 3730xl DNA analyzers (Macrogen, South Korea).

### Sequence alignment and phylogenetic reconstructions

Once the poriferan origin of the obtained sequences was verified using BLAST (http://blast.ncbi.nlm.nih.gov/Blast.cgi), sequences were aligned using Clustal W v.1.81 [51]. In cases where the forward and reverse reads do not match, we used BioEdit v.7.2.5 [52] and kept either the best quality of the two reads or introduced an IUPAC ambiguity code into the consensus sequence.

JModelTest 0.1.1 [53] was used to determine the best-fitting evolutionary model for each dataset. The model GTR+I+G was used for the mitochondrial and nuclear genes under the Akaike information criterium. Phylogenetic trees were constructed under Bayesian Inference (BI) and Maximum Likelihood (ML) criteria. BI analyses were performed with MrBayes 3.2.1 [54]. Four Markov Chains were run with one million generations sampled every 1000 generations. The chains converged significantly and the average standard deviation of split frequencies was less than 0.01 at the end of the run. Early tree generations were discarded by default (25%) until the probabilities reached a stable plateau (burn-in) and the remaining trees were used to generate a 50% majority-rule consensus tree. ML analyses were executed with PhyMLv3.0 program [55], [56]. We assessed the robustness of the tree clades in PhyML by a nonparametric bootstrap resampling with 1000 replicates.

Incongruence Length Difference (ILD) test (PAUP 4.0b10) was run [57] to verify sequence homogeneity or incongruence between the 18S and COI markers. The ILD test indicated no significant conflict (p = 0.93) between the two markers so a concatenated 18S-COI dataset was constructed with the species for which we had sequences for both markers.

### 18S rRNA secondary structure and morphological analysis

RNAfold web server (http://rna.tbi.univie.ac.at/cgi-bin/RNAfold.cgi) [58], [59] was used to determine the predicted secondary structure of the 18S V4 variable region for all species following Voigt et al. [60]. We used the default setting for all parameters except for the folding temperature. As the specimens came from different localities, we fixed the folding temperatures according to that of the specimen locality. However, the specimens belonging to *Geodia* spp., which were used as outgroups, lived in locations with contrasting temperatures. Only in this case, we used the default setting of 37°C [61]. The program automatically chooses the secondary structures with the lowest free energy (dG in kcal/mol) [60]. Following Gazave et al. [62], we encoded the different parts of the predicted secondary structures as elements and treated them as binary characters (presence/absence) in the morphological matrix. As the V4 of 18S secondary structure motifs were conserved across genera, according to the species sequenced, we assumed that the few species for which the 18S sequence was not available, shared the secondary structure motifs of the genus. The morphological/secondary structure matrix, consisting of 26 morphological characters and 13 18S secondary structure motifs, is made available in S1 File. A phylogenetic-tree was built with the morphological matrix under the maximum parsimony (MP) criterion using PAUP 4.0b10 [57] using a heuristic search and the branch swapping method with the tree bisection and reconnection (TBR) algorithm and ACCTRAN character-state optimization.

## Results

### Mitochondrial COI

**Fig 1:**
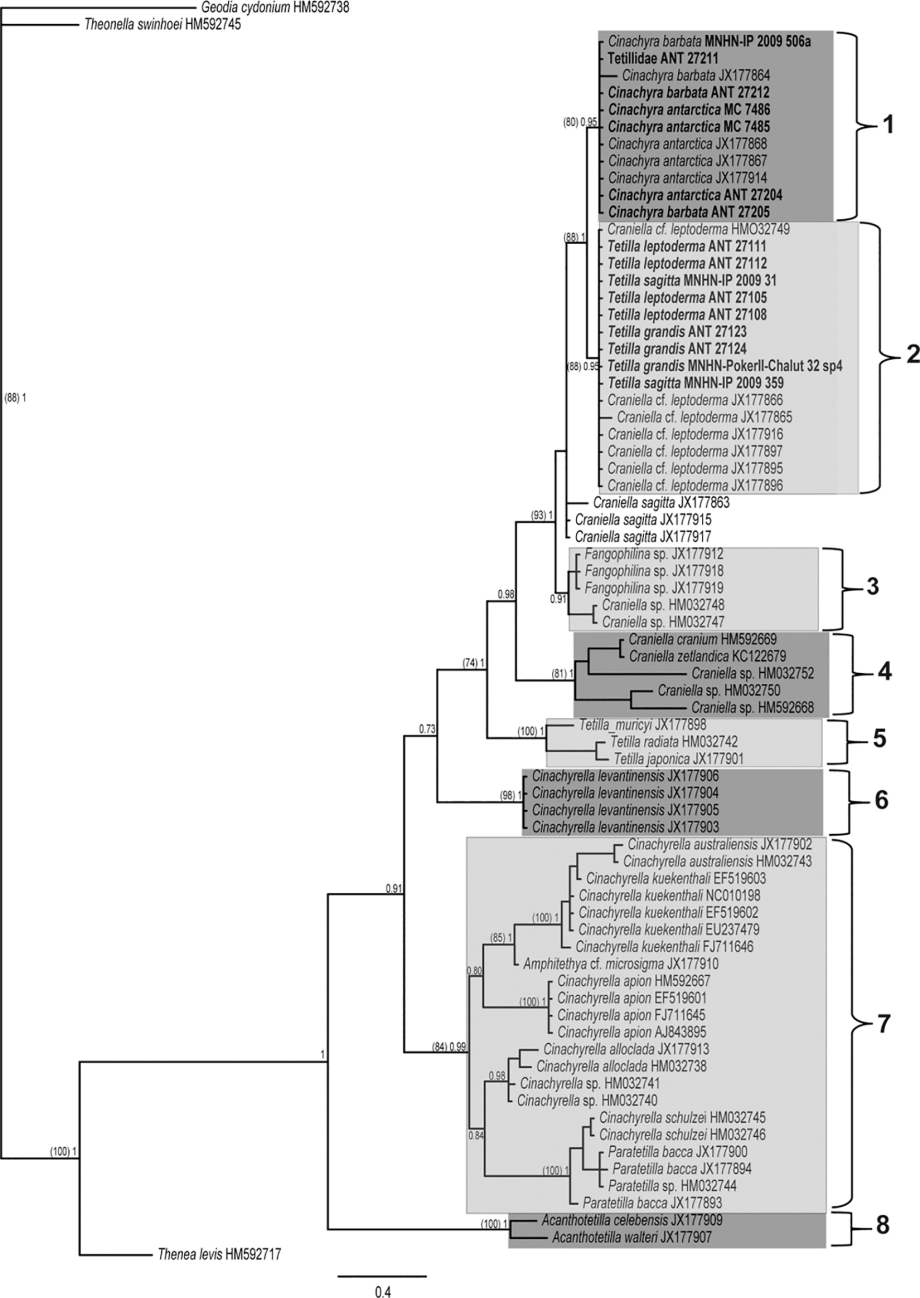
COI M1-M6, BI phylogeny of Tetillidae, which was congruent with ML tree. Species names are followed by their accession numbers (sequences downloaded from Genbank) or the specimen reference. Individuals sequenced in this study are in bold. Only supporting values higher than 70% (ML bootstrap, between parentheses on the left) or 0.75 (BI posterior probability) are represented on the tree nodes.

The COI (M1-M6 partition) dataset comprised 70 sequences (16 new) of 537 nucleotides (nt.) (158 nt. variable, of which 148 nt. were parsimony informative). Phylogenetic trees from ML and BI analyses retrieved congruent topologies, although some clades were differently supported (Fig 1). The Antarctic/New Zealand Tetillidae clustered in a well-supported group (93/1), which split in three well-defined clades henceforth called clades 1, 2 and 3.

Clade 1 (80/0.95, ML bootstrap supporting values /BI posterior probability) contained all the species of *Cinachyra* sequenced plus an unidentified species named Tetillidae ANT 27211.

Clade 2 (88/0.95) clustered individuals of *Tetilla grandis*, *T*. *sagitta*, and *T*. *leptoderma*, including those individuals called *Craniella leptoderma* in previous phylogenies. The position of three *Craniella sagitta* sequences (NIWA 25206, 28491, and 28929) was unresolved. These specimens were placed clearly apart from our *Tetilla sagitta* specimens from Antarctica, which morphologically conformed to the type species. Those three sequences belonged to two haplotypes with a difference of 8 nt.: NIWA 28491 and NIWA 25206 specimens from New Zealand *versus* NIWA 28929 from Antarctica.

Clade 3 (-/0.91) included the sequences of *Fangophilina* sp. (NIWA 28601, NIWA 28586, and NIWA 28617) and *Craniella* sp. (QMG 316342 and 316372), and was retrieved only in BI analyses.

Clade 4 (81/1) was a sister group of Antarctic sponges and included non-Antarctic/New Zealand species of *Craniella*.

Clade 5 (100/1) contained the non-Antarctic *Tetilla* (i.e. from tropical seas).

Clade 6 (98/1) included sequences of *Cinachyrella levantinensis* from the eastern Mediterranean, and was clearly apart from the rest of the *Cinachyrella* species.

Clade 7 (84/0.99) consisted of *Cinachyrella* species from tropical and subtropical waters and was divided in two well-supported sub-clades (posterior probability 0.80 and 0.84): the first included *C*. *australiensis*, *C*. *kuekenthali*, *Amphitethya* cf. *microsigma*, and *C*. *apion*, while the second included *C*. *alloclada*, *Cinachyrella* sp., *C*. *schulzei*, and *Paratetilla bacca*. The latter two species clustered together (100/1).

Clade 8 (100/1), included two species of *Acanthotetilla*, and appeared as the sister group of the remaining Tetillidae.

Almost no intra-species variation was found for the M1-M6 partition for the Antarctic genera, with the notable exception of two individuals: *Cinachyra barbata* (JX177864), which differed in 3 nt. from the other *Cinachyra* sequences, and *Craniella* cf. *leptoderma* (JX177865), which differed in 2 nt. from the other *Tetilla/Craniella* sequences (Fig 1).

**Fig 2:**
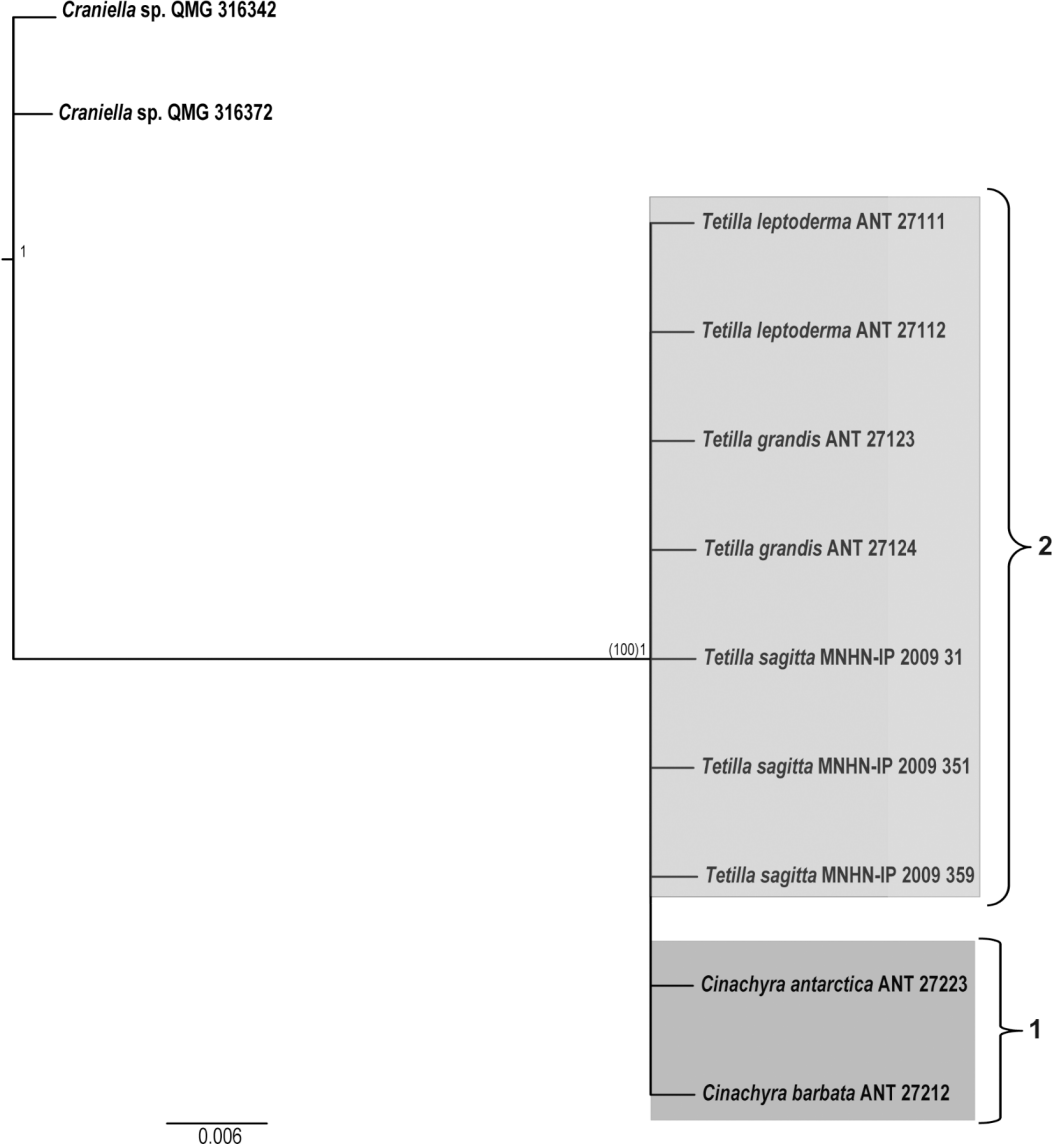
COI I3-M11 BI phylogeny of the Antarctic individuals of Tetillidae, which was congruent with ML tree, showing no clear separation between *Cinachyra* and *Antarctotetilla*. Bootstrapping and posterior probability (ML and BI, respectively) values are represented on the node of the only resulting clade. Individuals sequenced in the current study are indicated in bold.

We obtained 11 new sequences of the COI I3-M11 partition, 614 nt. long (11 nt. variable and parsimony informative). This partition (Fig 2), although it has been considered more variable than the M1-M6 partition [63], failed to reveal any difference among the Antarctic species of *Cinachyra* or *Tetilla*/*Craniella*.

### Nuclear 18S rRNA and 28rDNA

**Fig 3:**
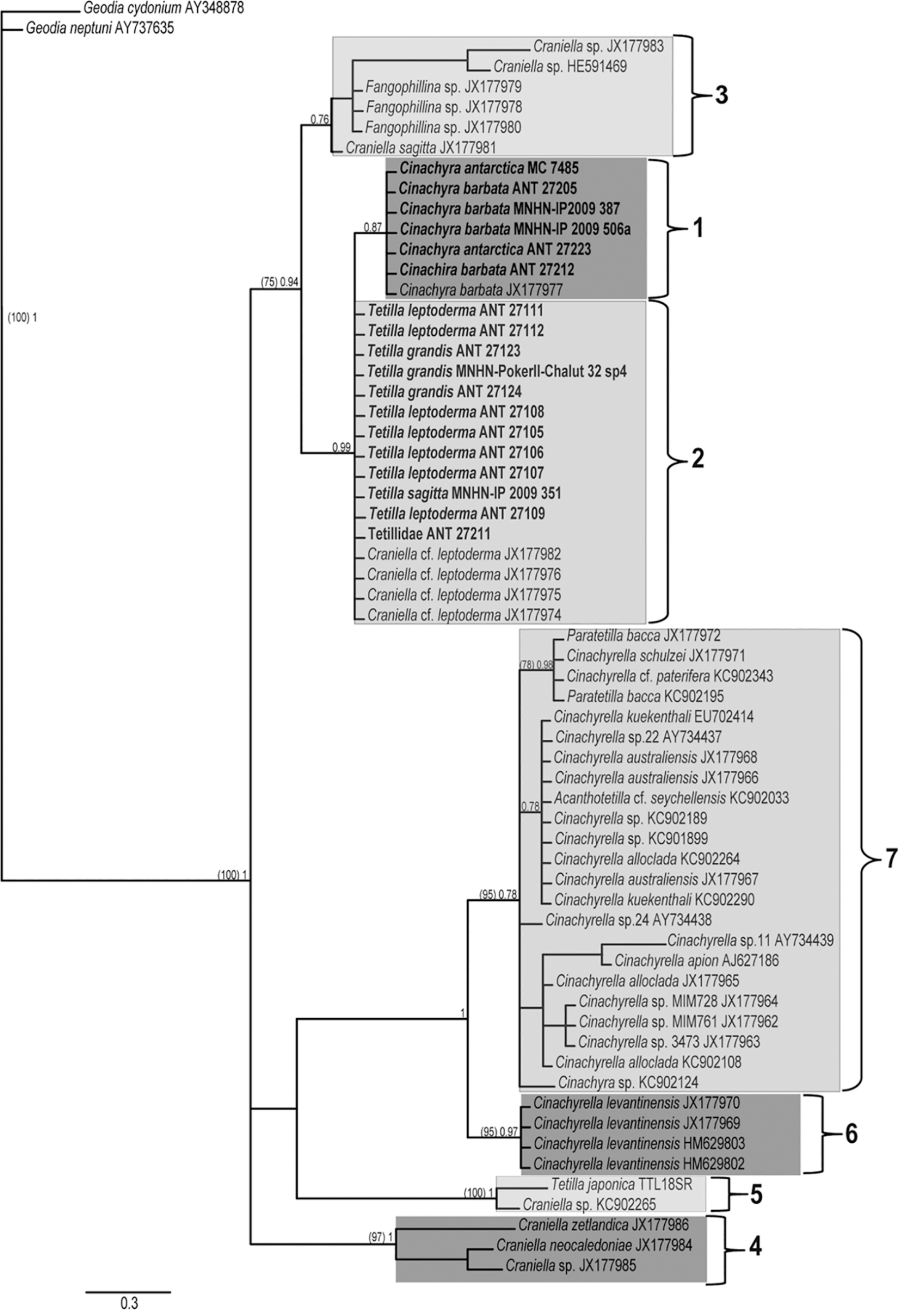
18S rRNA BI phylogeny of Tetillidae, which was congruent with ML tree. Species names are followed by the accession numbers (sequences downloaded from Genbank) or the specimen reference. Individuals sequenced in this study are in bold). Only supporting values higher than 70% (ML bootstrap, between parentheses on the left) or 0.75 (BI posterior probability) are represented on the tree nodes.

The full-length 18S dataset comprised 48 sequences (19 new) of 1483 nt. (83 nt. variable, of which 60 nt. were parsimony informative). ML and BI analyses gave congruent topologies (Fig 3). These trees recovered the same clades as the COI tree, except for the absence of clade 8, since no sequences of *Acanthotetilla* were available for this marker, and clade 2. Like in the COI analyses, 18S failed to discriminate among species of the Antarctic *Tetilla* (including species named *Craniella* in previous phylogenies) or *Cinachyra*. As in the COI phylogeny, the Antarctic/New Zealand Tetillidae clustered in a clade (75/0.94) comprising *Cinachyra* spp., *Tetilla* spp, *Craniella sagitta* (NIWA 25206), *Fangophilina* spp., and *Craniella* sp. (QMG 316342 and 316372) representatives.

Clade 1 (-/0.99) contained the species that split in clades 1 and 2 in COI phylogeny. The specimen Tetillidae ANT27211 groups this time with *Tetilla* spp. and not with *Cinachyra* spp., as in the COI phylogeny.

Clade 3 (-/0.76) comprises *Fangophilina* sp. plus *Craniella* sp. (QMG 316342 and 316372) and *Craniella sagitta* (NIWA 25206) as in the COI phylogeny but without statistical support.

Clade 4 (97/1) included the same *Craniella* species as in the COI phylogeny plus *C*. *neocaledoniae*, a species absent from the COI sampling.

Clade 5 (100/1) encompassed non-antarctic *Tetilla* (i.e. from tropical seas).

Clade 6 (95/0.97) was formed exclusively by *C*. *levantinensis* sequences. This clade was sister to clade 7 whereas it was sister to clade 1–5 in the COI tree.

Clade 7 (95/0.78), as in the COI tree, clustered all *Cinachyrella* species plus *Paratetilla bacca*.

**Fig 4:**
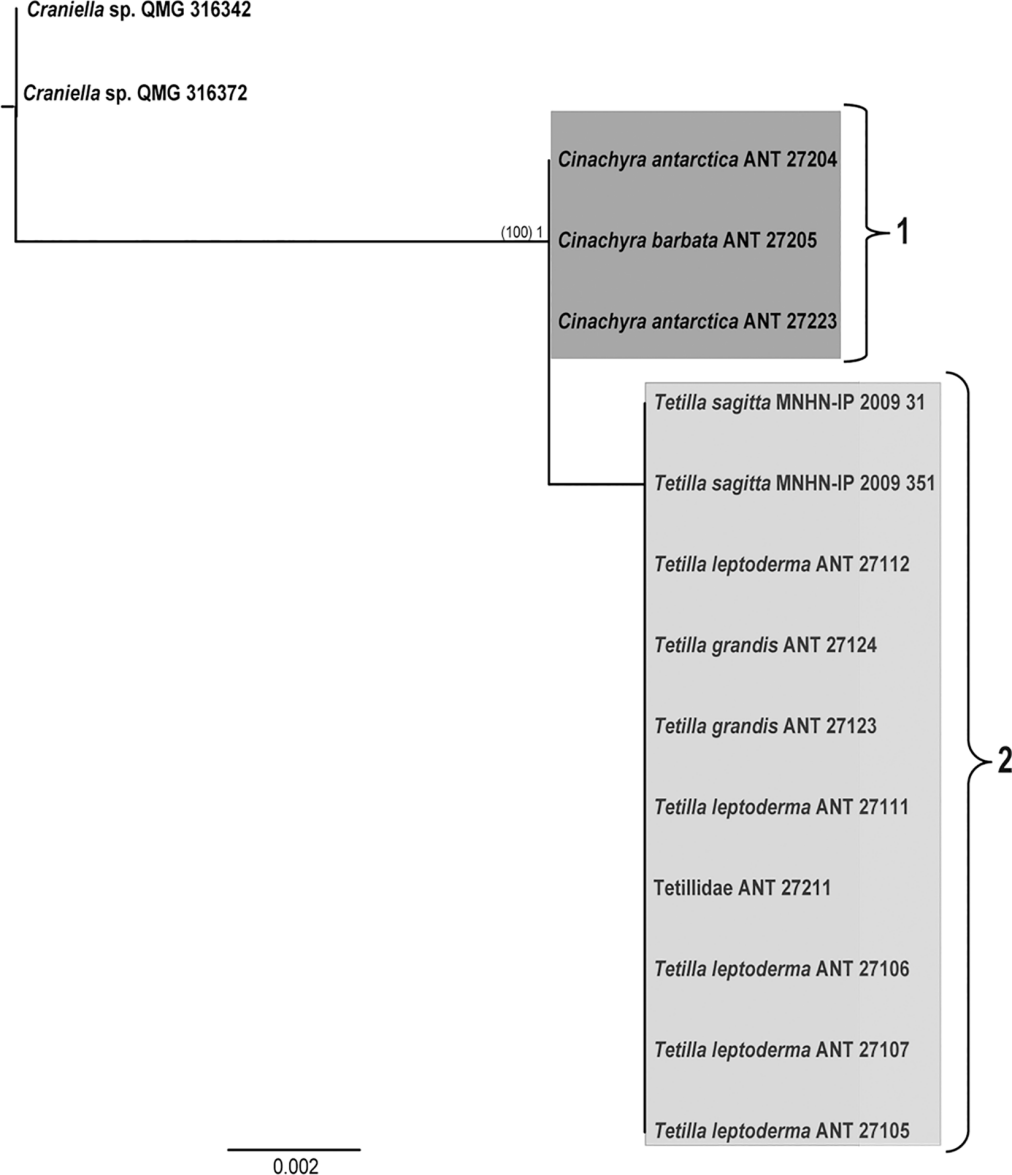
28S (D3-D5) BI and ML phylogeny of the Antarctic individuals of Tetillidae, which was congruent with ML tree, showing no species differences within the genera *Cinachyra* and *Antarctotetilla*. Species names are followed by the accession numbers (sequences downloaded from Genbank) or the specimen reference. Individuals sequenced in this study are in bold. Only supporting values higher than 70% (ML bootstrap, between parentheses on the left) or 0.75 (BI posterior probability) are represented on the tree nodes.

The 28S rRNA gene (D3-D5 partition) comprised 15 new sequences of 650 nt. (11 nt. variables, of which 10 nt. were parsimony informative). Phylogenetic trees were consistent in ML and BI analyses (Fig 4). Species within any of these two genera (*Cinachyra* and *Tetilla*/*Craniella*) were not discriminated.

### Concatenated COI and 18S rRNA

**Fig 5:**
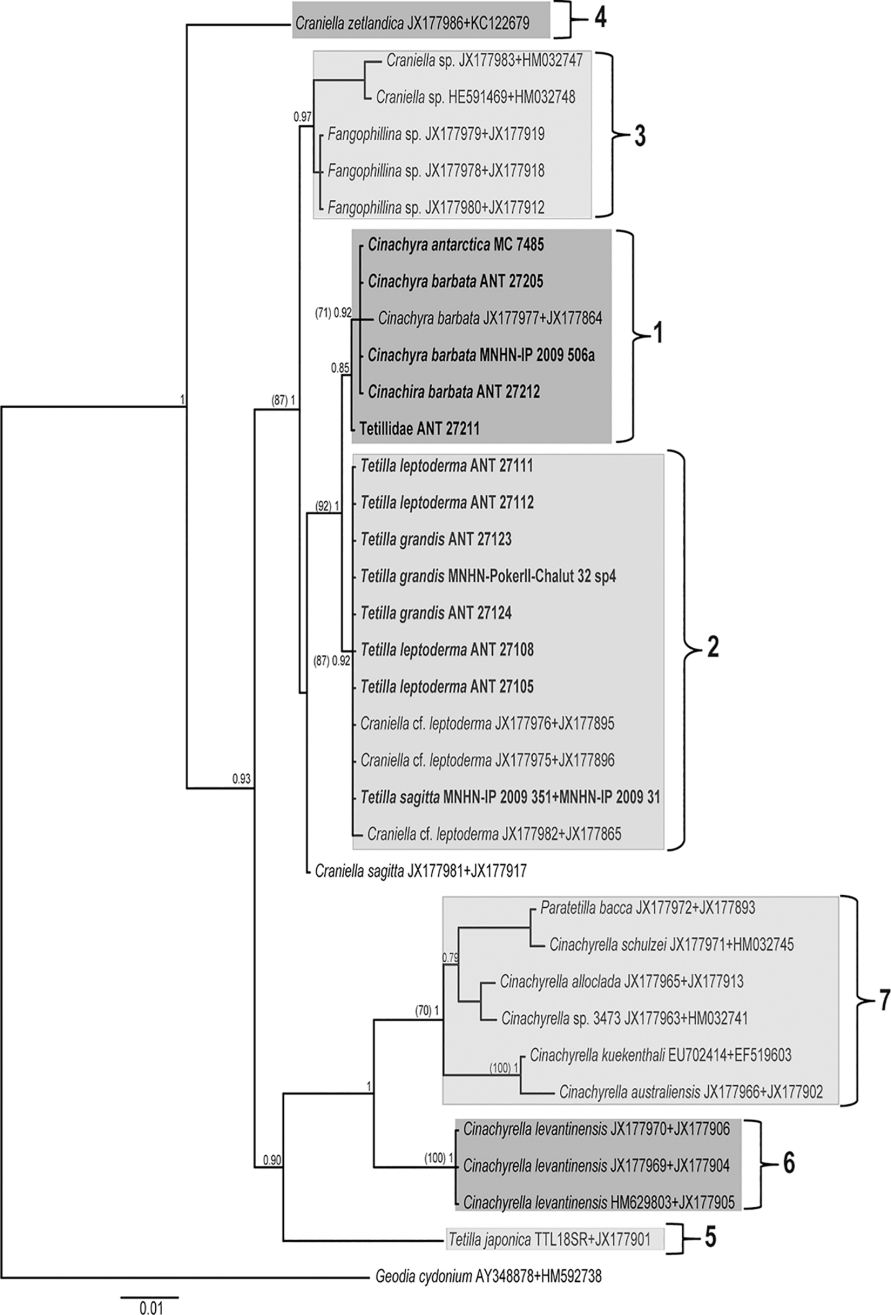
18S rRNA–COI M1-M6 concatenate BI phylogeny of Tetillidae, which was congruent with ML tree. Species names are followed by the accession numbers (sequences downloaded from Genebank) or the specimen reference. Individuals sequenced in this study are in bold. Only supporting values higher than 70% (ML bootstrap, between parentheses on the left) or 0.75 (BI posterior probability) are represented on the tree nodes.

The dataset for the concatenated mitochondrial and nuclear partitions (COI M1-M6 partition and 18S) comprised 39 sequences of 2019 nt. The resulting phylogenetic trees were consistent in ML and BI analyses (Fig 5) and were for the most part similar to the COI tree (i.e. clades 1, 3, 4, 5, and 7), except for clade 6, which was shared only with the 18S tree (Fig 3). On the other hand, contrarily to 18S phylogeny, clade 2 was well supported (87/0.92). Clade 3 was similar to both COI and 18S phylogenies. The supporting values of the clades slightly varied in some cases with respect to those of the previous phylogenies.

### Morphological identifications and re-examination of specimens

To understand our phylogenetic results, which were fairly congruent with both nuclear and mitochondrial genes, we examined the morphology of our specimens, several holotypes and also some specimens previously sequenced by Szitenberg et al. [34]. The resulting decisions from our examinations are detailed below and summarized in Table 1 and S2 File.

Clade 1 comprised individuals that belonged to the *Cinachyra* genus. Most of these individuals were morphologically similar except for the specimen of *C*. *barbata* (NIWA 28877), and that of *Cinachyra antarctica* (QMG 311149). The former differed from the other *C*. *barbata* in 3 nt, and pictures of the specimen (courtesy of M. Kelly) show porocalices spread on the sponge body instead of being concentrated on the lateral zone: we decided to name it *Cinachyra* cf. *barbata* until the specimen could be studied. Underwater pictures (courtesy of J. Hooper) of *Cinachyra antarctica* (QMG 311149) show a hairy surface covered with sediments with high palisades of spicules just around the openings: we tentatively renamed it as *Cinachyra* sp.

**Fig 6:**
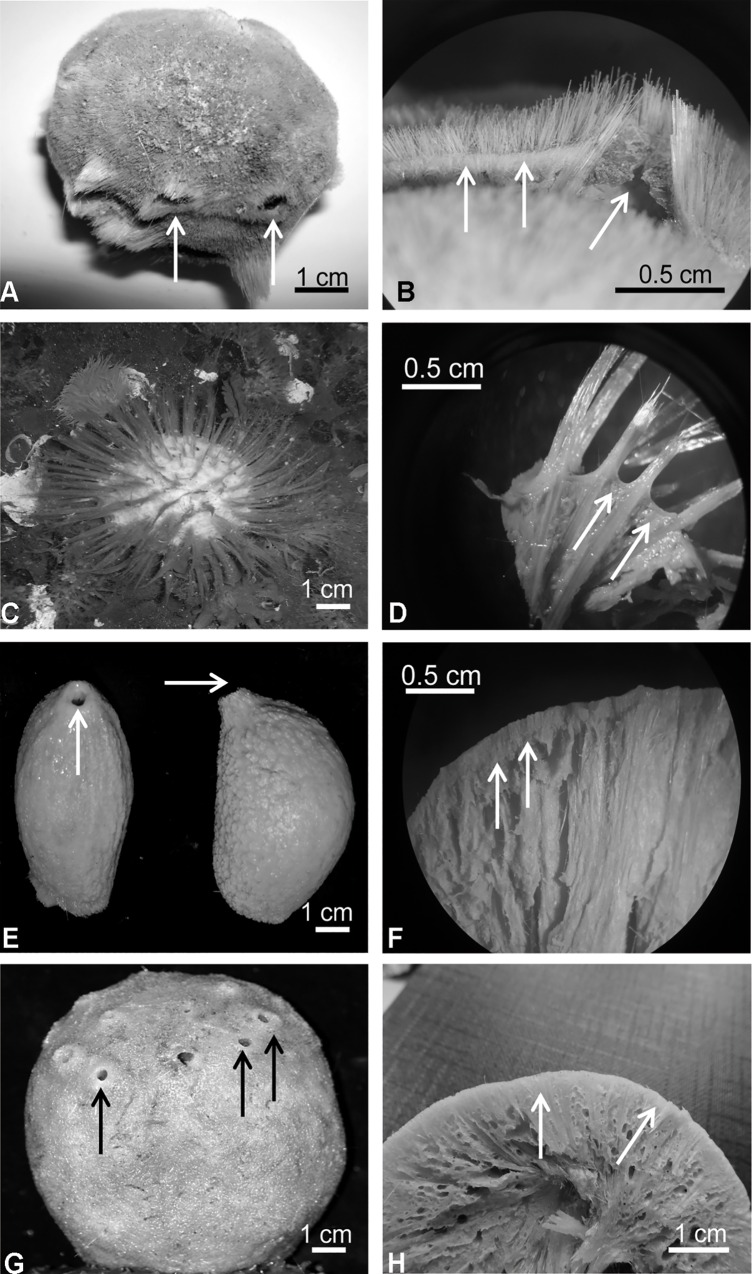
Pictures of the species of Tetillidae studied. A) *Cinachyra barbata* from Newmayer (Antarctica) arrows point to the porocalices. B) Transversal section of *C*. *barbata*: arrows point to the cortex and one porocalyx. C) Underwater picture of *Cinachyra antarctica* from McMurdo, (Antarctica). D) Transversal section of *C*. *antarctica*: arrows point to the collagenous cortex. E) *Antarctotetilla leptoderma* from South Georgia: arrows point to the unique osculum on top. F) Transversal section of *A*. *leptoderma*: arrows point to the dense ectosomal layer (pseudocortex). G) *Antarctotetilla grandis* from Newmayer, Antarctica: arrows point to the multiple oscula. H) Transversal section of *A*. *grandis*: arrows point to the slightly marked ectosomal layer. All the pictures are by the authors.

**Fig 7:**
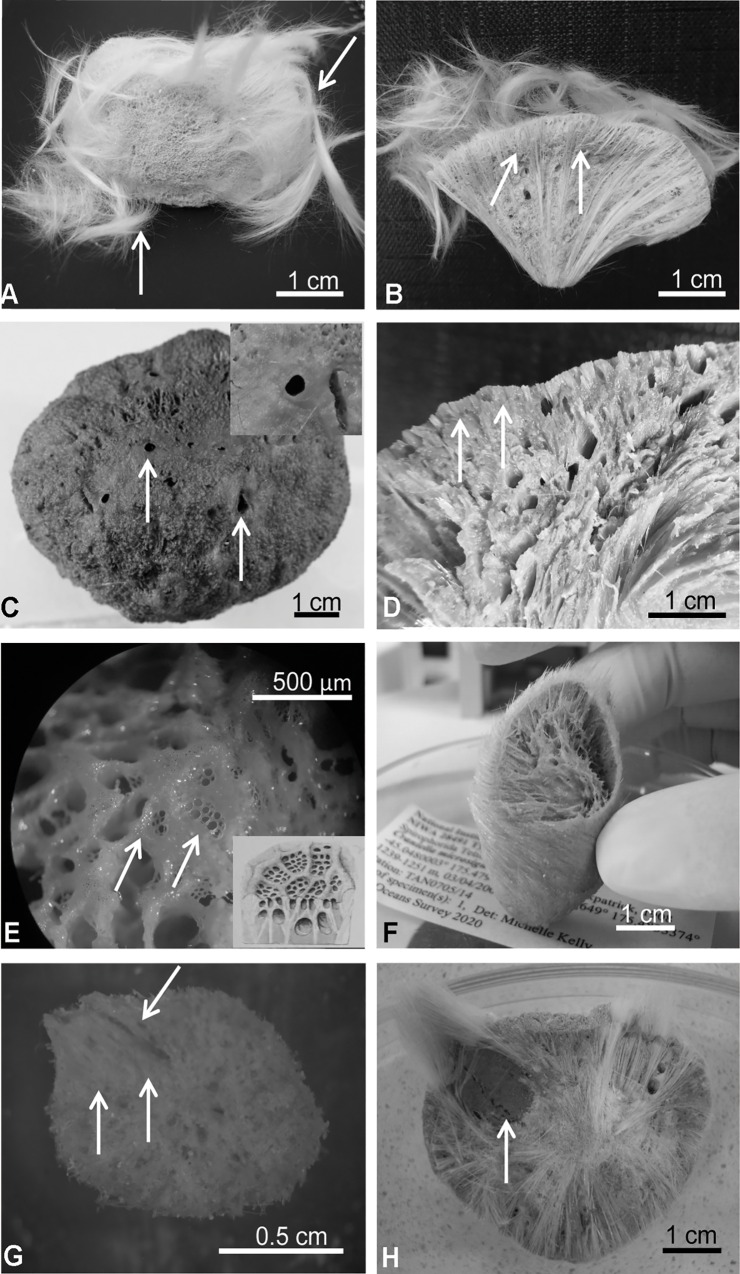
Pictures of the species of Tetillidae studied. A) Tetillidae ANT 27211 from Newmayer, Antarctica: arrows point to the hair-like hispidation pattern formed by bundles of fusiform oxeas, protriaenes and sometimes anatriaenes. B) Transversal section of Tetillidae ANT 27211: arrows point to the ectosomal layer. C) *Antarctotetilla sagitta* from Adélie Land: arrows point to the oscula; inset: detail of the even surface around the oscula. D) Transversal section of *A*. *sagitta*: arrows point to the ectosomal layer. E) Surface of *A*. *sagitta*: arrows point to the pores clustered in sieve-like areas; inset: *T*. *sagitta* pores in sieves by Kirkpatrick (1908). F) *Craniella sagitta* NIWA 28491 from New Zealand. G) cf. *Fangophilina* sp. NIWA 28601 from New Zealand: arrows point to the osculum. H) Lectotype of *Fangophilina submersa* MZSPO 160 from Northern Gulf of Mexico: arrow points to the porocalyx. All the pictures are by the authors except figures G, which were courtesy of Sadie Mills.

Clade 2 included *Tetilla*/*Craniella* specimens from Antarctica/New Zealand. These specimens belonged to three species (*leptoderma*, *sagitta* and *grandis*) that had been formally placed either in the genus *Tetilla* or the genus *Craniella* by previous authors. However, these species did not have the characteristic conspicuous double-layered cortex of *Craniella* [36]. Instead, they all had a loose arrangement of cortical oxeas perpendicular to the surface, which we will henceforth call ‘pseudocortex’ (Figs 6F and 6H and 7B and 7D). Therefore these species cannot belong to *Tetilla* either, which lacks a cortical specialization. Moreover, all these species have pores clustered in small, depressed areas and their oscula, single or multiple, are usually larger than in typical *Craniella*. Finally, they never harbored direct developing embryos as observed in both typical *Craniella* and some *Tetilla*. All these characteristics prompted us to erect a new genus for these three species: *Antarctotetilla* gen. nov. (see definition below). We will henceforth call these species *Antarctotetilla leptoderma*, *Antarctotetilla sagitta* and *Antarctotetilla grandis*. The latter had been synonymized with *A*. *leptoderma* [14], [22] but it is clearly different from *A*. *leptoderma* as it has several small oscula and a spherical body while *A*. *leptoderma* is slightly elongate/ovoid body with a sole large oscule on top. Therefore we propose to officially resurrect this species so far only recorded from the Antarctic and Sub-Antarctic.

Five out of the eight specimens of *Craniella* cf. *leptoderma* from Szitenberg et al. [34] were confirmed morphologically to be *A*. *leptoderma*. Pictures of NIWA 28910 and NIWA 36097 showed that these specimens had a smooth surface and multiple small oscula, which matched the morphology of *Antarctotetilla grandis*. Finally, underwater pictures of QMG 315031 showed that the specimen had at least two oscula, which differs from the large single oscule on top, constantly present in *A*. *leptoderma*. QMG 315031 was therefore tentatively re-identified as *Antarctotetilla* cf. *sagitta*.

The three specimens of *Craniella sagitta* (NIWA 28491, NIWA 25206, and NIWA 28929 (Table 1) were examined (see pictures of NIWA 28491 in Fig 7F) and compared with the syntype of *Tethya sagitta* (ZMB Por 3504). These three specimens possessed a pseudocortex but lacked the main diagnostic characters of the species, such as pores grouped in sieve-like areas and oscula on the top of smooth flattened zones (Fig 7C and 7E) [16]. Thus, they cannot be identified with *A*. *sagitta* but we are unsure to which species or even genera they should belong. We could distinguish two morphotypes that corresponded to two haplotypes (differing in 8 nt.), which suggests that they represent two different species, here named Tetillidae sp. 1 and 2. Interestingly, these specimens were originally identified as two different species based on external morphology (S2 File).

**Fig 8:**
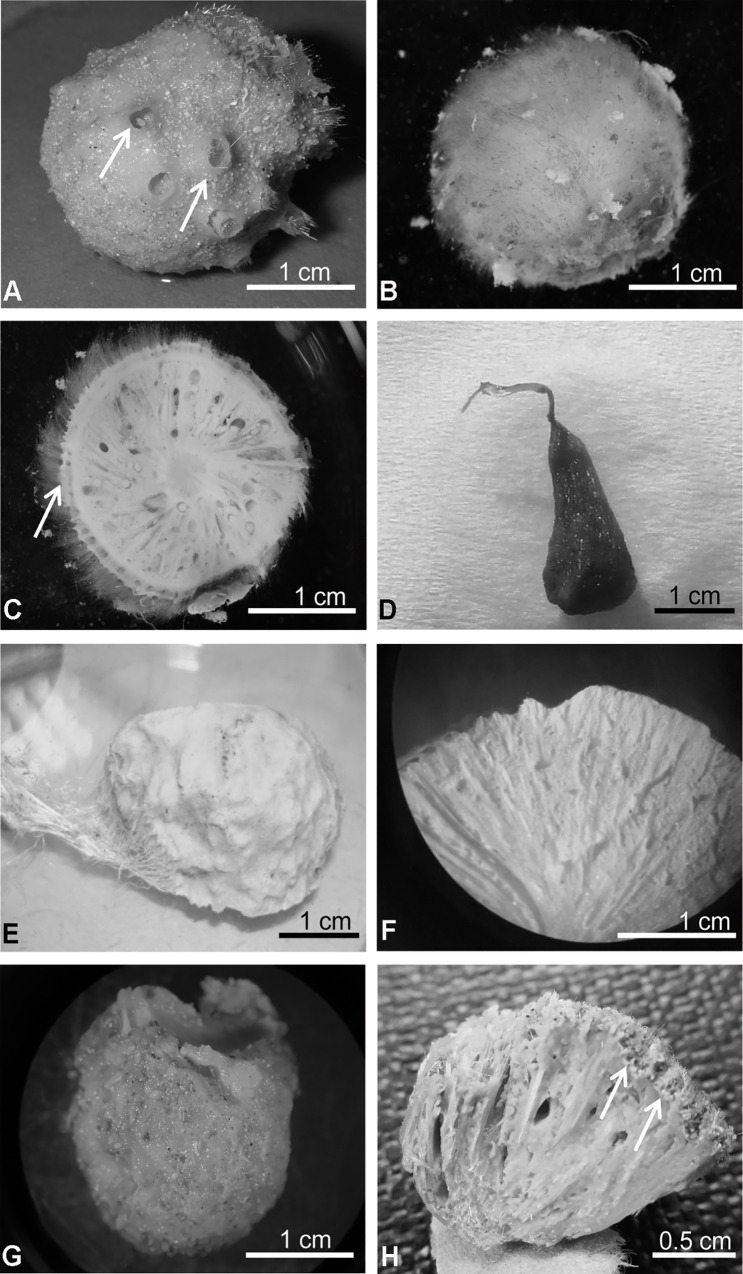
Pictures of the species of Tetillidae studied. A) *Craniella* sp. QMG 316342 from Australia: arrows point to the porocalices. B) *Craniella* cf. *cranium* ZMBN 85240 from Norway. C) Transversal section of *Craniella* cf. *cranium* ZMBN 85240: arrow points to the double-layered cortex. D) Holotype of *Tetilla euplocamos* MZSPO 206 from Brazil. E) Paratype of *Tetilla muricyi* UFBA 2569 from Brazil. F) Transversal section of the paratype of *T*. *muricyi* UFBA 2569. G) *Levantinella levantinensis* from Lebanon. H) Transversal section of *L*. *levantinensis*: arrows point to the dense ectosomal region formed by sediment accumulation. All the pictures are by the authors except figures A, and D, which were courtesy of John Hooper, and Marie Meister, respectively.

Clade 3 included two misidentified genera: *Craniella* sp. (QMG 316342 and QMG 316372) and *Fangophilina* sp. (NIWA 28601, NIWA 28586, and NIWA 28617). The individuals called *Craniella* sp. from the Norfolk Ridge (Fig 8A), had porocalices and a spiculous cortex, similar to that of *Cinachyra*. They are therefore tentatively identified as *Cinachyra* sp. As for *Fangophilina* sp., its morphology was different from the species type of *Fangophilina*, *F*. *submersa* (Fig 7H). However, the small size of these specimens (ca. 1cm in diameter) prevented us to verify whether the only visible orifice was a true porocalyx or an oscule and thus they have been provisionally named cf. *Fangophilina* sp.

**Fig 9:**
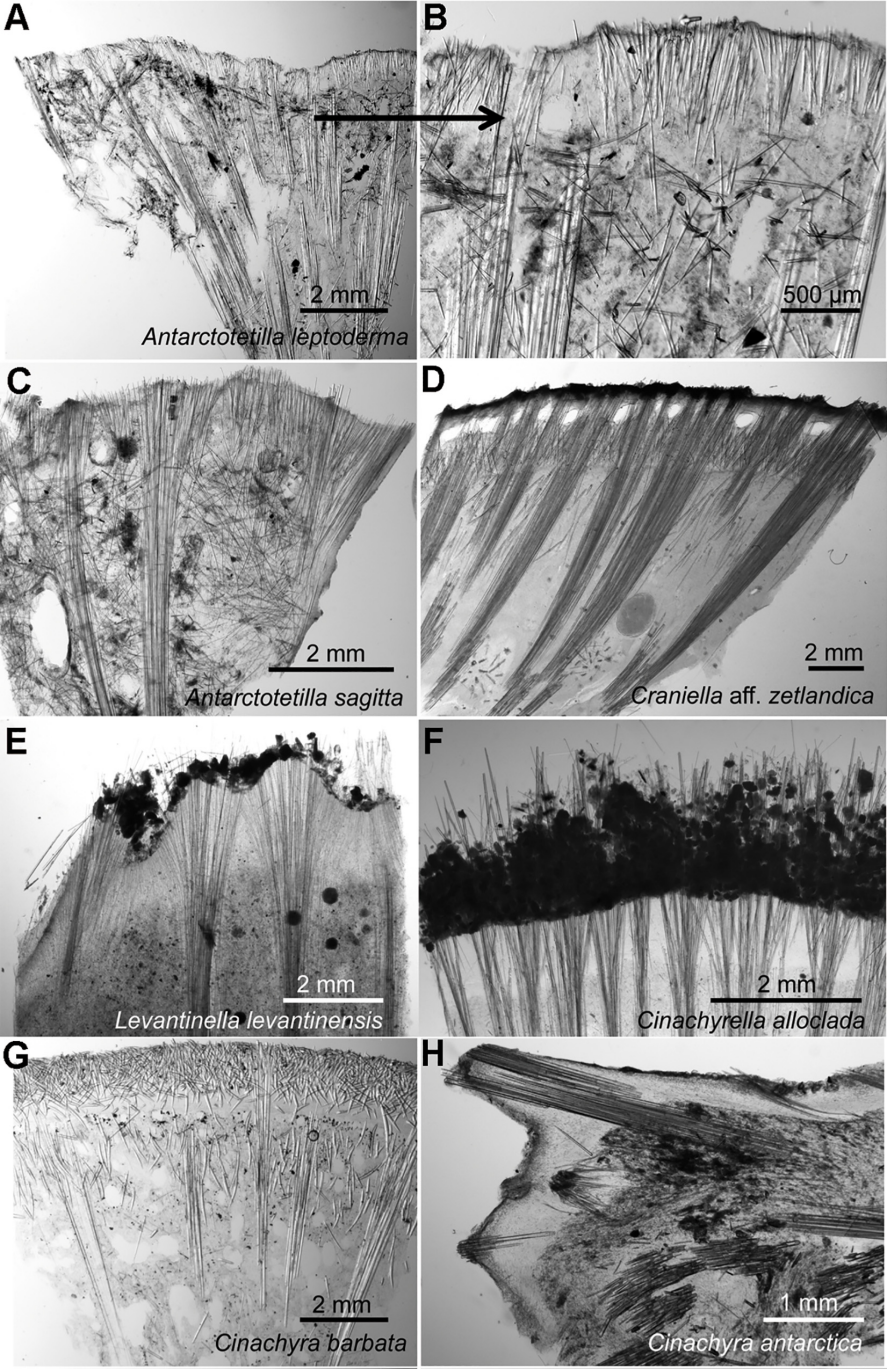
Thick sections of Tetillidae species showing differences in the ectosome or cortex structures. A) *Antarctotetilla leptoderma* from Adélie Land, Antarctica, MNHN IP-2009-544a. B) *A*. *leptoderma*, close-up of C. C) *Antarctotetilla sagitta* from Adélie Land, Antarctica, MNHN IP-2009-31. D) *Craniella* aff. *zetlandica* from Korsfjord, Norway, ZMBN 85239. E) *Levantinella levantinensis* from Israel, PC 705. F) *Cinachyrella alloclada* from Bocas del Toro, Panama, ZMBN 81788. G) *Cinachyra barbata* from Adélie Land, Antarctica, MNHN IP-2009-387. H) *Cinachyra antarctica* from Adélie Land, Antarctica, MNHN IP-2009-328.

Clade 4 included *Craniella* species from the Boreo-Arctic Atlantic and the Norfolk ridge. We confirm that they possessed the typical double-layered cortex of *Craniella* (Fig 9D), and were thus considered true *Craniella*. *Craniella cranium* Müller, 1776 (ZMBN 85239) and *Craniella* sp. (ZMBN 85240) from Korsfjord-Norway [64] were reexamined. ZMBN 85239 was re-identified as *Craniella* aff. *zetlandica* since it only differed from *C*. *zetlandica* Carter, 1872 in the presence of sigmaspires. ZMBN 85240 was re-identified as *Craniella* cf. *cranium* because it closely resembled *Craniella pilosa* Montagu, 1818, a synonym of *C*. *cranium* (Fig 8B and 8C). However, we keep the ‘cf.’ for now, until a world-wide revision of *C*. *cranium* can be made, this species having a long and complicated taxonomic history.

Clade 5 contained *Tetilla* species, which are characterized by the absence of a true cortical structure. Re-examination of a picture of the type species, *T*. *euplocamus* (Fig 8D), and the types of *T*. *muricyi* Fernandez, Peixinho, Pinheiro, and Menegola, 2011 (Fig 8E and 8F) and *T*. *radiata* Selenka, 1879, under the stereomicroscope seems to confirm previous studies of *Tetilla radiata* Santos and Hajdu, 2003 (Figs 5 and 6), and *T*. *muricy* Fernandez et al. 2011 (Fig 4C and 4D), which showed the absence of a cortical specialization in these *Tetilla*. However, a loose layer of para-tangential large oxeas below the surface may be present in some cases, as it has been reported for *T*. *rodriguezi* Fernandez et al. 2011 (Fig 6C).

Clade 6 only contained *Cinachyrella levantinesis*. We re-examined nine specimens of *C*. *levantinensis* trying to understand why they did not group with any of the other *Cinachyrella* species in the molecular trees. *C*. *levantinensis* lacks cortex as *Cinachyrella* (Figs 8H and 9E). Its surface is mostly covered with a dense layer of sand, which was retained by the protruding spicules (Fig 8G) as in some *Cinachyrella* (Fig 9F). However, while *Cinachyrella* species have typical large porocalices, *C*. *levantinensis* has numerous small rounded depressions, difficult to assign to porocalices with certainty. As stated by Vacelet et al. [65], these depressions were not visible in specimens heavily covered by sand. These depressions are sometimes concentrated in sand free lateral areas, whereas usually porocalices are evenly distributed in typical *Cinachyrella*. All these characteristics prompted us to officially create *Levantinella* gen. nov. (see definition below) to welcome this species.

Clade 7 contained only specimens of *Cinachyrella*. Some individuals of dubious identification were revised (S2 File). *Amphitethya* cf. *microsigma* (SAM-S1189) from Szitenberg et al. [34] showed an external morphology and spicules corresponding to the original description of *A*. *microsigma* Lendenfeld, 1907. Furthermore the individual SAM-S1189 was collected in southern Australia, not far from the type locality of *A*. *microsigma*. We therefore confirmed the species identification. Using the online SpongeMaps (http://www.spongemaps.org), we also examined underwater pictures of *Cinachyrella schulzei* Keller, 1891 (QMG 320143, QMG 320636) from Szitenberg et al. [41, 34]. These specimens are reddish pink (QMG 320636) to pale pink (QMG 320143), and completely devoid of sand, in contrast to the typical yellowish-grey color of *C*. *schulzei*, which is often covered with sand [66]. These specimens may instead be conspecific with *Cinachyrella tenuiviolacea* Pulitzer-Finali, 1982, which has a typical pink color and is fairly common in Australian shallow waters and were here provisionally referred to as *C*. cf. *tenuiviolacea*.

### Maximum parsimony phylogeny on phenotypic characters

**Fig 10:**
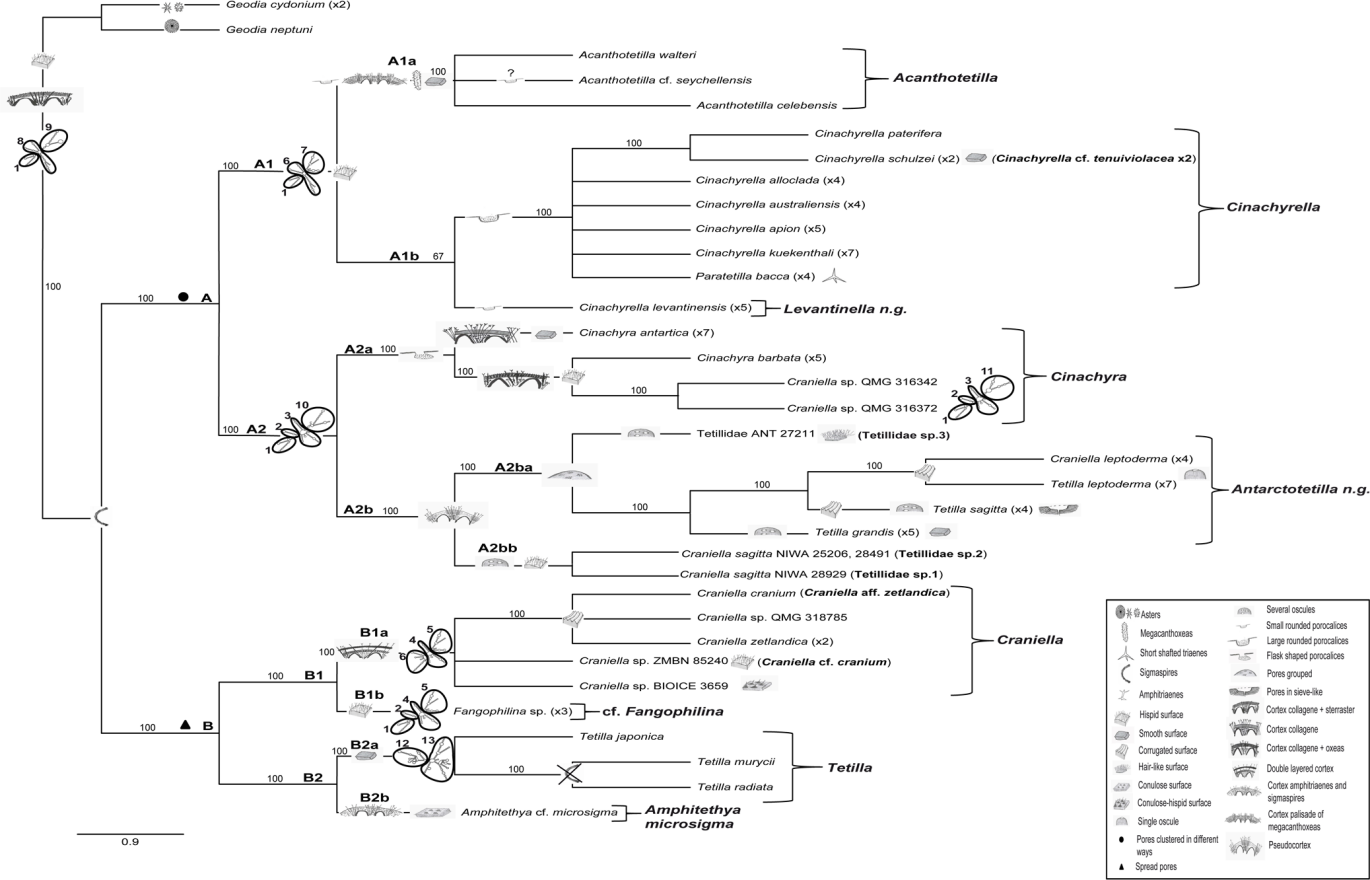
**A) Phylogram of one of the most parsimonious trees on morphological characters plus the several zones identified for the V4 region of 18S secondary structure–SSRs–(numbered and encircled)**. Characters that represent either synapomorphies or apomorphies are depicted in the tree. The supporting bootstrap values of clades resulting from the Majority-rule consensus tree are also indicated. **B)** legends to character drawings on the tree.

The MP analysis included 33 OTUs, one for each species, 26 morphological characters, and 13 motifs of the V4 18S secondary structure (S3 File), which were treated as binary elements (S1 File). The astrophorin *Geodia cydonium* and *Geodia neptuni* were used as outgroups. The MP analysis retrieved 6 most parsimonious trees of 48 steps (CI = 0.684; RI = 0.887). Character states are shown at the nodes of the phylogram corresponding to tree number 1 (Fig 10). Clade support resulting from the majority-rule consensus tree is also shown on the phylogram (Fig 10).

The Tetillidae appeared divided in two strongly supported clades (100% bootstrap value), here called clade A and B. Clade A was characterized by pores grouped in several ways and was formed by two well supported sub-clades: A1 embraced two groups with the same V4 secondary structure, A1a formed by *Acanthotetilla*, with acanthoxeas, small porocalices, and a cortical region made of a palisade of acanthoxeas. The clade A1b was formed by the genus *Cinachyrella* and *Levantinella* gen. nov. The latter species is characterized by absence of a well-formed cortex, small, little conspicuous porocalice-like structures, and a dense incorporation of sand to its surface. The genus *Cinachyrella* included *Paratetilla bacca* despite the presence of triaenes in the former, and was characterized by the absence of a defined cortex and the presence of true hemispherical large porocalices. No morphological characters could differentiate the two subgroups of *Cinachyrella* found in the molecular phylogenies (i.e. the *C*. *australiensis*/*C*. *apion* group versus the *C*. *alloclada*/*C*. cf. *tenuiviolacea* group).

A2 corresponded to the Antarctic clade, characterized by sharing the same V4 secondary structure. A2a included the *Cinachyra* genus, with cortex and flask-shaped porocalices as synapomorphies, and two *Cinachyra* sp. (QMG 316342 and QMG 316372), which also have cortex and porocalices but show a slightly different secondary structure. Clade A2b, with pseudocortex as a synapomorphy (Fig 9A, 9B and 9C), was subdivided in 2 groups: A2ba and A2bb. A2ba included *Antarctotetilla* gen. nov. with pores clustered in shallow small depressions as a synapomorphy, and Tetillidae sp.3 (ANT 27211, Figs 7A and 9B) totally covered by long hair-like spicules, without porocalices, and with pores clustered in shallow small depressions. A2bb comprised Tetillidae sp. 1 and sp. 2 individuals (NIWA 28491, 25206 and 28929).

Clade B included species that had spread pores as a synapomorphy and was also formed by two well-supported sub-clades:

B1 included two groups. Group B1a was formed by species of *Craniella* with a two-layered cortex made of collagen plus spicules, and a hispid-conulose surface, and a specific V4 secondary structure. Group B1b included cf. *Fangophilina sp*, with a particular V4 secondary structure region and a hispid surface (Fig 7G).

B2 was divided in two groups: B2a formed by the genus *Tetilla* (tropical species) with a particular V4 secondary structure and B2b containing *Amphitethya microsigma*.

Species of the genera *Cinachyra* and *Antarctotetilla* gen. nov. were clearly differentiated, despite the fact that they shared the same V4 secondary structure: *Cinachyra antarctica* has a collagen cortex as an autopomorphy (Figs 6C and 6D and 9H) while *C*. *barbata* and the two *Cinachyra* sp. (QMG 316342 and QMG 316372) have a spicule-collagenous cortex (Figs 6A and 6B and 9G).

All molecular analyses suggested that the latter species was related to cf. *Fangophilina* sp. and not to *Cinachyra*. Within *Antarctotetilla* gen. nov., *A*. *leptoderma* and *A*. *sagitta* have a corrugate surface (Figs 6E and 7C), but while the former has a single large apical osculum, *A*. *sagitta* shows several small oscules (Fig 7C). On the other hand, *A*. *grandis* shows an even surface, spread small oscula, and pores clustered in small depressions (Fig 6G), while *A*. *sagitta* is characterized by their pores in sieves covering sub-ectosomal spaces (Fig 7C and 7E).

### Formal diagnoses of the currently proposed genera

Family Tetillidae

*Tetilla* Schmidt, 1868

Type species: *Tetilla euplocamos* Schmidt, 1868.

Diagnosis: Tetillidae without porocalices, without cortical specialization, without auxiliary megascleres [36].

*Cinachyrella* Wilson, 1925

Type species: *Tetilla hirsuta* Dendy, 1889.

Diagnosis: Tetillidae usually with hemispherical porocalices (except in some stalked species), without spiculous cortical specialization; modified from [36].

Remarks: *Amphithetya* and *Paratetilla* are within the *Cinachyrella* clade in our pylogenies. The several well-supported subgroups within the *Cinachyrella* clade might correspond to sub-generes. However, a more in deep study of *Cinachyrella* by including additional species would be necessary to formally propose those subgeneres.

*Levantinella* gen. nov.

Type species: *Levantinella levantinensis* (Vacelet et al., 2007) by monotypy.

Diagnosis: Tetillidae with small porocalices formed by rounded shallow depressions, without cortex, without auxiliary megascleres.

*Craniella* Schmidt, 1870

Type species: *Craniella tethyoides* Schmidt, 1870.

Diagnosis: Globular sponges without porocalices and with a distinct, two-layered cortex (visible to the naked eye). The outer cortex layer often with sub-dermal cavities and the inner layer made of collagen and a tight arrangement of cortical oxeas. Presence of direct-developing embryos within the sponge tissue; modified from [36].

*Cinachyra* Sollas, 1886

Type species: *Cinachyra barbata* Sollas, 1886.

Diagnosis: Tetillidae with a collagenous cortex, sometimes reinforced by auxiliary oxeas, with flask-shaped porocalices; modified from [36].

Remarks: Most described *Cinachyra* have been transferred to *Cinachyrella* (see World Porifera Dadabase)

*Antarctotetilla* gen. nov.

Synonymy: *Tetilla sensu* Sollas, 1886 (part.), *Craniella sensu* Kirkpatrick, 1908 (part.).

Type species: *Tetilla leptoderma* Sollas, 1886 by designation.

Diagnosis: Tetillidae with single or multiple oscule(s) on top, pores clustered in shallow depressions (no porocalices) and a pseudocortex (very slight cortical differentiation) made of perpendicular oxeas, loosely arranged.

Remarks: Our molecular and morphological results suggest that that three species should be assigned to this genus: *A*. *leptoderma*, *A*. *sagitta*, and *A*. *grandis*. These species are only known (up to now) from the Antarctic, New Zealand, Kerguelen Islands and the Magellanic region. Moreover, *Craniella coactifera* Lendenfeld, 1907 shows strong similarities with *A*. *grandis* and is likely a synonym of this species. We reallocate *C*. *coactifera to Antarctotetilla* gen. nov. and keep this species valid until its type can be compared with the type of *A*. *grandis*.

## Discussion

### Molecular markers

The molecular markers used in this study were informative enough to resolve the relationships of Tetillidae genera but did not resolve Antarctic species. Phylogenetic trees inferred with the COI M1-M6 partition (also known as the barcoding Folmer fragment) gave a better resolution of the genera, in part because of the higher number of individuals sequenced for this marker and those already available in Genbank. However, although the COI M1-M6 partition differentiated all the species of temperate and tropical genera of Tetillidae included in this study it failed to separate species within the Antarctic genera *Cinachyra* and *Antarctotetilla* gen. nov., despite clear morphological differences. Although uncommon, strictly identical COI M1-M6 sequences for different sponge species have previously been found in other demosponge groups [67], [68], [69], [70] and also in Antarctic hexactinellid sponges: only two COI haplotypes were found among the Antarctic *Rosella* species [71], which had been recognized by Barthel and Tendal [72].

Similarly, the 18S, 28S (D3–D5) and COI I3-M11 partitions did not succeed in discriminating all Antarctic species. This was to be expected from the slow evolving 18S or even from the faster evolving 28S (D3-D5), which has rather been used for inter-species relationships. However, this was rather surprising for the COI I3-M11 partition, which is considered more variable than the Folmer partition [63], and has been used in population genetics and phylogeography studies of demosponges [73], [48], [74].

This may be an indication of contrasting evolutionary rates between sponge groups [75], [69]. Thus, our results suggest either a particularly slow genetic evolutionary rate of the markers 28S (D3-D5) and COI or a recent radiation with phenotypic characters evolving faster than the genes studied. Further studies on other Demospongiae families and/or more variable markers are required to shed light on the evolutionary processes that affect Antarctic sponges, which are poorly studied so far.

### Phylogeny and taxonomic actions

Insufficient knowledge on the morphological characters of Antarctic/New Zealand Tetillidae along with misidentifications biased the interpretation of previous phylogenetic studies [34]. The re-examination of some of these specimens as well as holotypes (S2 File) proved essential to understand previous puzzling results such as the polyphyly of *Craniella* [34]. Overall, our results improve and clarify the Tetillidae phylogeny. All COI and 18S trees, as well as the COI+18S trees were mostly congruent, except for the positions of Tetillidae sp.3 (ANT 27211), Tetillidae sp.1 (“*Craniella sagitta”* NIWA 28929), Tetillidae sp.2 (“*Craniella sagitta”* NIWA 25206 and 28491) and *Levantinella levantinensis*.

The MP trees allowed us to identify phenotypic synapomorphies for the proposed genera. The MP phylogeny on phenotypic characters, plus the motifs of the 18S secondary structure (V4 region), differed from the molecular phylogenies only in the position of the two *Cinachyra* sp. (QMG 316342 and QMG 316372, previously wrongly identified as *Craniella* sp.): they form a strongly supported clade with cf. *Fangophilina* sp. (NIWA 28601, 28586, 28617) in molecular trees, whereas they group with *Cinachyra* in the MP tree (because of their porocalices and spiculous cortex).

According to Szitenberg et al. [34], the presence of cortex or/and porocalices, which have been traditionally used to differentiate Tetilllidae genera [35], [36], do not represent synapomorphies according to our molecular analyses. Instead, the types of cortex (with spicules, without spicules, with one or two layers) and/or of porocalices (e.g. deep flask-shaped, hemispherical porocalices, and small, shallow cavities) are the derived characters shared within each genus.

In the current study, we see the Tetillidae basically divided in seven well-supported clades, instead of the five recovered in Szitenberg et al. [34]. Most of these clades correspond to genera: *Antarctotetilla* gen. nov., *Cinachyra*, *Acanthotetilla*, *Tetilla*, *Cinachyrella*, *Craniella*, and *Levantinella* gen. nov.

The new genus *Antarctotetilla* contains exclusively those Tetillidae without cortex, without porocalices, and with grouped ostia. The presence of a pseudocortex (not visible with the naked eye) instead of a clearly conspicuous cortex in those species may explain why they have been assigned to the genus *Tetilla* by previous authors [76], [77], [19], [22]. However, the type species of *Tetilla* (*T*. *euplocamos* Schmidt, 1868) and other tropical representatives that we have examined (e.g. *T*. *radiata*, *T*. *muricyi*), do not have any kind of obvious cortical specialization.

After moving the traditional Antarctic “*Tetilla*” (*Craniella* in Szitenberg et al. [34]) to *Antarctotetilla* gen. nov., the genus *Tetilla* recovers its classical diagnosis [35] by including those Tetillidae without cortex and without porocalices. Similarly, by moving the two misidentified *Craniella* sp. (QMG 316342 and QMG 316372) to *Cinachyra* sp., *Craniella senso stricto* [35], [36] was recovered as a monophyletic genus, with a characteristic two-layered cortex as synapomorphy.

Szitenberg et al. [34] proposed the inclusion of *Fangophilina* and *Cinachyra* within the genus *Craniella*, as either junior synonyms or sub-genera. However, this proposal was based on a series of misidentifications: QMG 316342 and QMG 316372 had been wrongly identified as *Craniella* sp. (now *Cinachyra* sp.), NIWA 28929, NIWA 28491 and NIWA 25206 had been wrongly identified as *Craniella sagitta* (here renamed Tetillidae sp. 1 and sp. 2). Conversely four confirmed *A*. *sagitta* specimens (ANT IP 31, IP 351, IP 359 and *A*. cf. *sagitta* QMG 315031) joined the *Antarctotetilla* gen. nov. clade in both molecular and morphological phylogenies.

We missed true *Fangophilina* species in our molecular analyses, since, as stated above, the three cf. *Fangophilina* sp. did not completely match the diagnostic morphological characters of the genus. The type species *F*. *submersa* was reported to have two opposite porocalyces: one with an inhalant function and the other exhalant [78], [36]. The revision of the type material showed that only the cavity containing the ostia is a true porocalyx since the other one corresponded to a deep cloacal osculum. Morphological and genetic investigations on further individuals of *F*. *submersa*, which is known just by a single specimen, are necessary to resolve the phylogenetic position and morphological variation of this genus, which has been considered dubious [36].

Our phylogenetic trees retrieved the type species of *Amphitethya* (*A*. *microsigma*) and *Paratetilla* (*P*. *bacca*) within the large *Cinachyrella* clade, as in previous phylogenies [34]. The position of *Paratetilla* within *Cinachyrella* is also recovered in the morphological tree as species of both genera have similar external morphology. Although the type species of *Cinachyrella*, *C*. *hirsuta* Dendy, 1889, is not included in our sampling, we are confident that it would group in the large *Cinachyrella* clade, based on its morphology [36]. We would therefore have enough arguments to synonymize *Amphitethya* Lendenfeld, 1907 and *Cinachyrella* Wilson, 1925 with *Paratetilla* Dendy, 1905, the oldest genus name. However, reallocating the so far described 40 species of *Cinachyrella* [33] to *Paratetilla* without previous reexamination would not be the most conservative option since it would hide the morphological difference we currently recognize (calthrop-like triaenes) to identify *Paratetilla*. Instead, we prefer to wait for further molecular phylogeny studies on this group to take taxonomic action. Since *Cinachyrella* species are distributed in several clades, we believe a future revision of this group with a wider sampling might indicate where to place *C*. *hirsuta*.

The presence of *Amphitethya* within the *Cinachyrella* clade of our molecular trees was unexpected, due to its stalked morphology and the absence of porocalices, which, conversely, placed *A*. *microsigma* as a sister species of *Tetilla* in the morphological tree. However, we note that the characteristic amphitriaenes of *Amphitethya* have also been occasionally observed in *Paratetilla* species: *P*. *aruensis* [79], and *P*. *merguiensis* [80].

In our 18S phylogeny, a few sequences from Redmond et al. [39] had suspicious phylogenetic affinities: *Acanthotetilla* cf. *seychellensis* (KC902033), and *Cinachyra* sp. (KC902124) clustered with *Cinachyrella* while *Craniella* sp. (KC902265) clustered with *Tetilla*. We suspect these are misidentifications but we could not re-examine the corresponding specimens.

Szitenberg et al. (S3 File) [34] suggested erecting *Levantinella* gen. nov. for the species *C*. *levantinensis*, which substantially diverged from the rest of the *Cinachyrella* species in their study. However, no formal definition was proposed thus making *Levantinella* a *nomen nudum*. We agree with these authors in that this species belongs to a new genus and we formally propose to make *Levantinella* gen. nov. available with *C*. *levantinensis* as type species by monotypy. The phylogenetic affinities of *Levantinella*, however, differed depending on the gene partition used: it appeared as a sister group of the *Tetilla/Craniella/Fangophilina/Antarctotetilla/Cinachyra* clade with COI while it was a sister group to the other *Cinachyrella* with 18S.

The family Tetillidae appeared paraphyletic or polyphyletic in previous 18S phylogenies [39], [38] and 28S phylogenies [81]: the *Craniella/Cinachyra/Antarctotetilla* gen. nov. clade was sister to the Astrophorina. However, these results may be due to a sampling bias, a possibility further suggested by a wide COI phylogeny that recovers a monophyletic Tetillidae [38]. A more thorough worldwide study of representatives of this family is needed to further test its monophyly.

### Geographical distribution of Tetillidae genera

**Fig 11:**
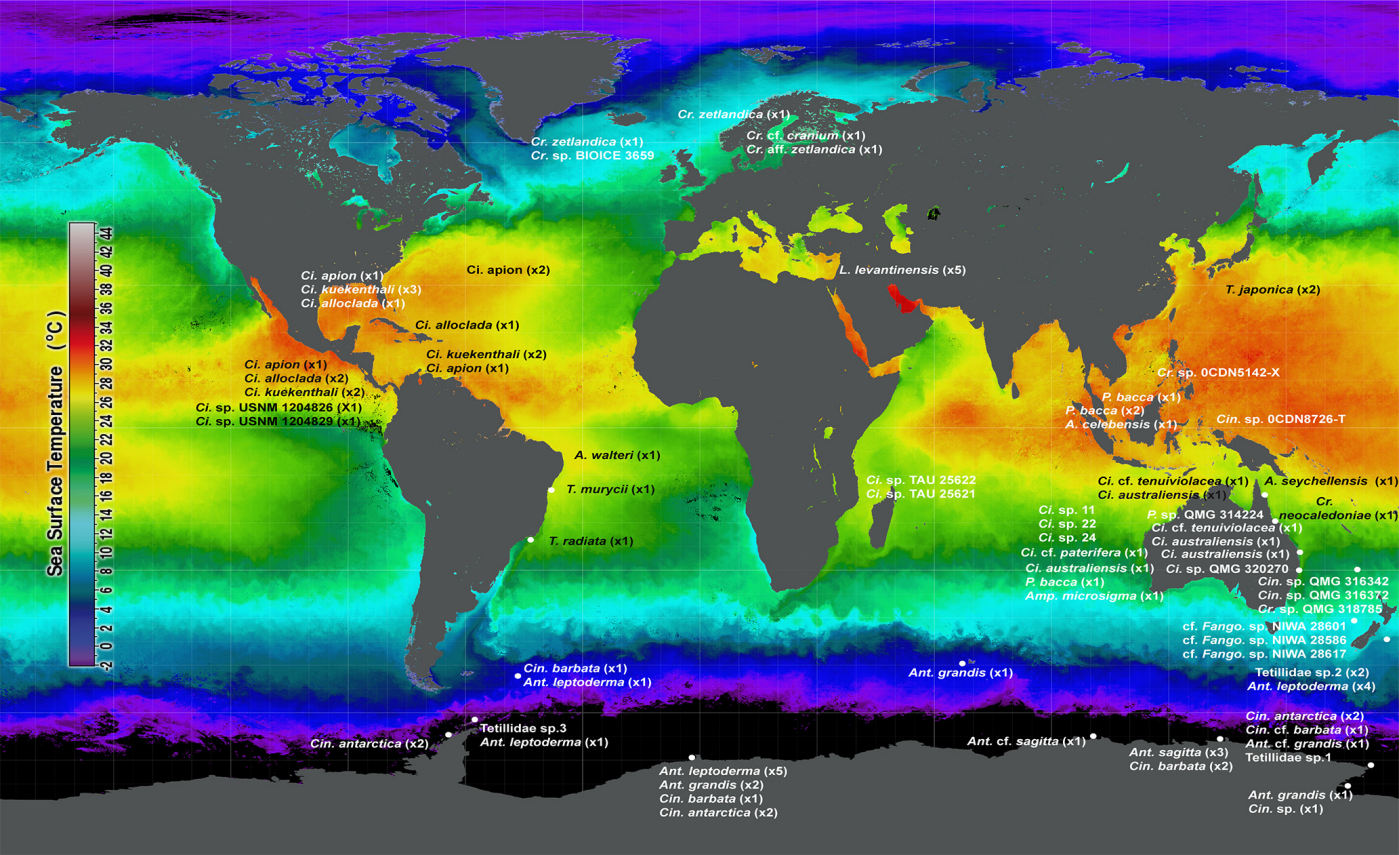
Distribution of the Tetillidae species analysed in this study overlying a temperature map in South hemisphere winter (NASA Goddard Space Flight Center, Ocean Ecology Laboratory, Ocean Biology Processing Group; (2014): Sea-viewing AQUA MODIS Sea Surface Temperature, August 2013. NASA OB.DAAC. http://oceancolor.gsfc.nasa.gov/cgi/l3. Accessed on: 2015/04/29). White points represent exact sampling locations. Cr. = *Craniella*; Ci. = *Cinachyrella*; L. = *Levantinella; Fango*. = *Fangophilina*; A. = *Acanthotetilla*; T. = *Tetilla*; Amp. = *Amphitethya*; P = *Paratetilla*; Cin. = *Cinachyra*; Ant. = *Antarctotetilla*.

The geographical location of the studied Tetillidae suggests a temperature related distribution of some genera (Fig 11). *Cinachyrella* shows a tropical–subtropical distribution, while *Craniella* species mainly inhabit temperate-cold seas. The genera *Cinachyra* and *Antarctotetilla* appear confined to the Antarctic and Sub-Antarctic regions, contributing to the reported Antarctic sponge endemism [82], [5], which underlines the importance of the Polar Front in isolating the Southern Ocean fauna. Other *Cinachyra* species, which have been reported out of the Antarctic, may have been incorrectly attributed to this genus. For example, *Cinachyra helena* Rodriguez and Muricy, 2007 from Brazil does not belong to *Cinachyra* since its purported porocalyx rather looks like a cloacal oscula with macro-orifices inside the depression [83]. Moreover, its two-layered cortex [83] suggests that it is a *Craniella*. We therefore propose to reallocate this species to the genus *Craniella*. Other described *Cinachyra* have been moved to *Cinachyrella* posteriorly (see WPD). Current representatives of the genus *Tetilla* are mainly living in arctic-temperate-warm seas.

**Fig 12:**
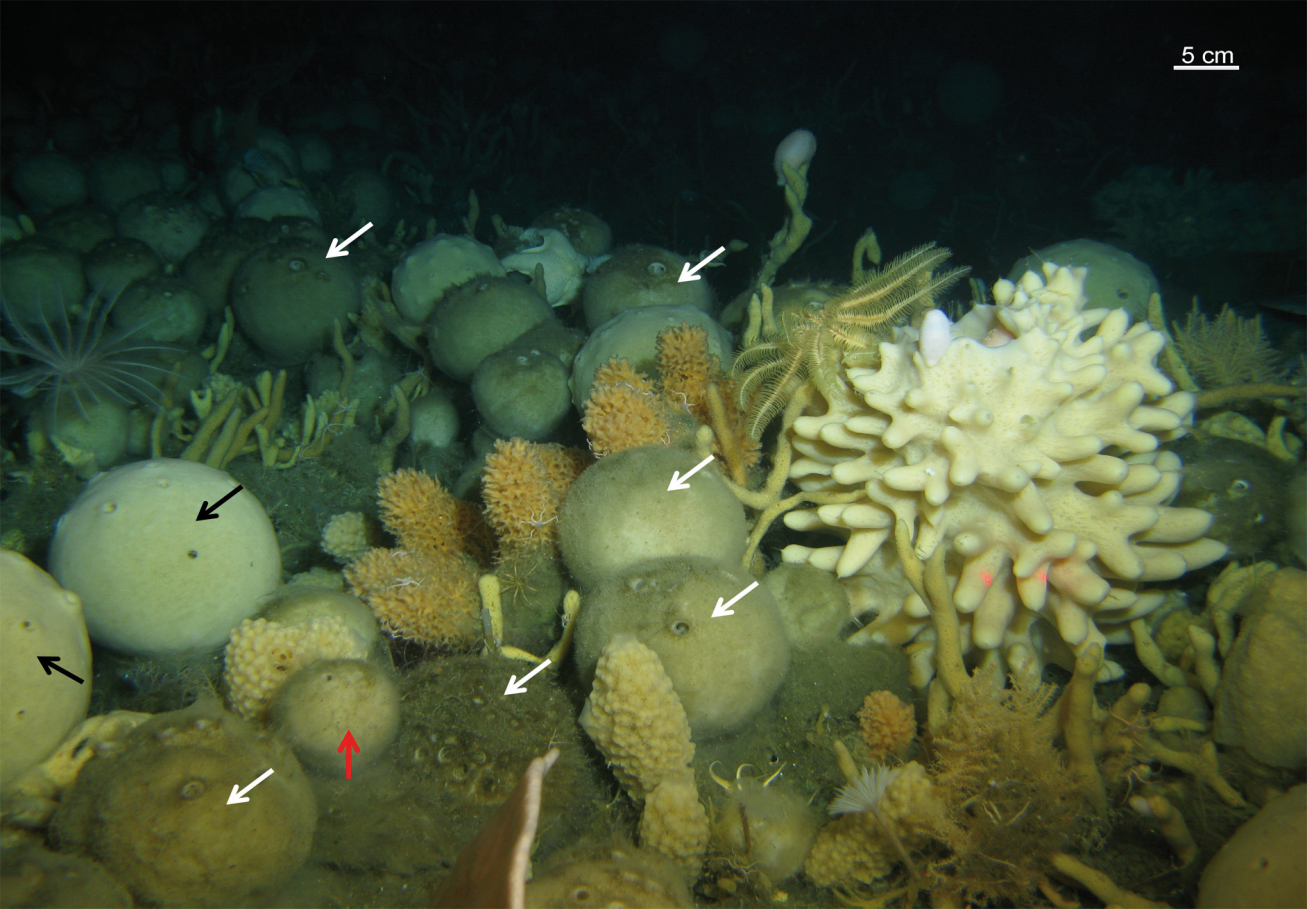
Tetillidae grounds on the Antarctic bottoms. Black arrows point to *Antarctotetilla grandis*. White arrows indicate *Cynachyra* spp. Red arrow points to Tetillidae sp3.

In relation to depth, *Cinachyrella* is distributed in shallow-waters (<30 m depth) with few exceptions that can be found up to 100 m depth (e.g. *Cinachyrella kuekenthali*, *Amphitethya microsigma*) while other genera such as *Craniella* or *Fangophilina* are nearly restricted to the deep-sea. *Antarctotetilla* and *Cinachyra* are eurybathic inhabiting from 30 to 600 m of depth, but they are particularly abundant between 200 and 300 m of depth where they may dominate in sponge grounds (Fig 12).

Sponge molecular phylogenies have greatly contributed to emend the traditional sponge systematics, in particular for demosponges [50], [84]. The sponge phylogenetic tree resolution continuously improves as new genetic markers, and more importantly, additional taxa are included in the datasets. However, a careful morphological identification of the individuals sequenced and included in the molecular phylogenies is required for a precise interpretation of the molecular results. In other words, molecular phylogenies should consistently be associated with thorough morphological studies of the specimens sequenced, as we have tried to do with the Tetillidae species.

## Supporting Information

S1 File Morphological data matrix (including secondary structure shapes of the 18S V4 variable region) used for the maximum parsimony phylogeny. (PDF)

S2 File Original and revised identifications of Tetillidae voucher specimens from previous studies (Cárdenas et al. 2011, Szitenberg et al. 2010, Szitenberg et al. 2013) after morphological re-examination. Localities, depths, and Genbank accession numbers of the corresponding sequences are also listed. QMG, Queensland Museum, Brisbane, Australia; NIWA, National Institute of Water & Atmospheric Research, New Zealand; SAM, South Australia Museum; ZMBN, Zoological Museum in Bergen, Norway. In bold, specimens re-examined in this study. Specimens with (*) were only seen on pictures.
(PDF)

S3 File The different parts of the predicted secondary structures (V4 region of 18S) are encircled and numbered. (PDF)
