# Correction: Phylogenetic Reassessment of Antarctic Tetillidae (Demospongiae, Tetractinellida) Reveals New Genera and Genetic Similarity among Morphologically Distinct Species

**DOI:** 10.1371/journal.pone.0167011

**Published:** 2016-11-16

**Authors:** Mirco Carella, Gemma Agell, Paco Cárdenas, Maria J. Uriz

There is a spelling error throughout the manuscript. The spelling for the new genus *“Levantinella*” is incorrect. The correct spelling should be “*Levantiniella*.”
