# Supplementary material for: Phylogenetic Reassessment of Antarctic Tetillidae (Demospongiae, Tetractinellida) Reveals New Genera and Genetic Similarity among Morphologically Distinct Species
Source: PLoS One. 2016 Aug 24;11(8):e0160718. doi: 10.1371/journal.pone.0160718 (PMC4996456; doi:10.1371/journal.pone.0160718)
Supplement: S1 File — (PDF) [file pone.0160718.s001.pdf]

Phylogenetic reassessment of Antarctic Tetillidae (Demospongiae, Tetractinellida) reveals new genera and genetic similarity among morphologically distinct species

Carella M[1], Agell G[1], Cárdenas[2],[3]P, Uriz MJ[1]*

[1]Centre d'Estudis Avançats de Blanes (CEAB-CSIC). Accés Cala St Francesc 14. 17300 Blanes (Girona) Spain

[2] Département Milieux et Peuplements Aquatiques, Muséum National d'Histoire Naturelle, UMR 7208 "BOrEA", Paris, France

[3]Department of Medicinal Chemistry, Division of Pharmacognosy, BioMedical Centre, Husargatan 3, Uppsala University, 751 23 Uppsala, Sweden

* Corresponding author: iosune@ceab.csic.es

S2 Table. Morphological data matrix (including secondary structure shapes of the 18S V4 variable region) used for the maximum parsimony phylogeny.

NAME	single oscule	multioscule	Big Hemisferical porocalices	small rounded porocalices	flask shaped porocalices	Pores grouped	Pores Sieve-like	Pores not Grouped
Acanthotetilla celebensis (de Voogd & van Soest, 2007)	?	?	0	1	0	0	0	0
Acanthotetilla walteri (Peixinho, Fernandez, Oliveira, Caires & Hajdu, 2007)	?	?	0	1	0	0	0	0
Acanthotetilla seychellensis (Thomas, 1973)	?	?	0	?	0	0	0	0
Cinachyra antartica (Current study)	?	?	0	0	1	0	0	0
Cinachyra barbata (Current study)	?	?	0	0	1	0	0	0
Tetillidae ANT 27211 (Current study)	0	1	0	0	0	1	0	0
Cinachyrella alloclada (Uliczka, 1929)	?	?	1	0	0	0	0	0
Cinachyrella paterifera (Wilson, 1925)	?	?	1	0	0	0	0	0
Cinachyrella australiensis (Carter, 1886)	?	?	1	0	0	0	0	0
Cinachyrella levantinensis (Vacelet, Bitar, Carteron, Zibrowius & Perez, 2007)	?	?	0	1	0	0	0	0
Cinachyrella schulzei (Keller, 1891)	?	?	1	0	0	0	0	0
Craniella cranium (Müller, 1776)	?	?	0	0	0	0	0	1
Craniella cf. *leptoderma* (Szitenberg, 2013)	1	0	0	0	0	1	0	0
Craniella sp. QMG 316342 (Szitenberg, 2013)	?	?	0	0	1	0	0	0
Craniella sp. QMG 316372 (Belinky, 2012)	?	?	0	0	1	0	0	0
Craniella sp. ZMBN 85240 (Cardenas, 2011)	?	?	0	0	0	0	0	1
Craniella sp. BIOICE 3659 (Szitenberg, 2010)	?	?	0	0	0	0	0	1
Craniella sp. QMG 318785 (Szitenberg, 2013)	?	?	0	0	0	0	0	1
Craniella zetlandica (Carter, 1872)	?	?	0	0	0	0	0	1
Cinachyrella kuekenthali (Uliczka, 1929)	?	?	1	0	0	0	0	0
Fangophilina sp. (Szitenberg, 2013)	?	?	0	0	0	0	0	1
Geodia cydonium (Jameson, 1811)	?	?	0	0	0	0	0	1
Geodia neptuni (Sollas, 1886)	?	?	0	0	0	0	0	1
Cinachyrella apion (Uliczka, 1929)	?	?	1	0	0	0	0	0
Paratetilla bacca (Selenka, 1867)	?	?	1	0	0	0	0	0
Tetilla leptoderma (Current study)	1	0	0	0	0	1	0	0
Tetilla grandis (Current study)	0	1	0	0	0	1	0	0
Tetilla japonica (Lampe, 1886)	?	?	0	0	0	0	0	1
Tetilla murycii (Peixinho, Pinheiro & Menegola, 2011)	?	?	0	0	0	0	0	1
Tetilla radiata (Selenka, 1879)	?	?	0	0	0	0	0	1
Tetilla sagitta (Cardenas, 2008)	0	1	0	0	0	1	1	0
Amphitetya microsigma (Lendenfeld, 1907)	?	?	0	0	0	0	0	1
Craniella sagitta (Szitenberg, 2013)	0	1	0	0	0	0	0	1

NAME	CORTEX only colagene	Double layered CORTEX	CORTEX colagene + oxeas	CORTEX colagene + sterraster	CORTEX palisade of megacanthoxeas	CORTEX amphitriaenes and sigmaspires	No CORTEX	Pseudocortex
Acanthotetilla celebensis (de Voogd & van Soest, 2007)	0	0	0	0	1	0	0	0
Acanthotetilla walteri (Peixinho, Fernandez, Oliveira, Caires & Hajdu, 2007)	0	0	0	0	1	0	0	0
Acanthotetilla seychellensis (Thomas, 1973)	0	0	0	0	1	0	0	0
Cinachyra antartica (Current study)	1	0	0	0	0	0	0	0
Cinachyra barbata (Current study)	0	0	1	0	0	0	0	0
Tetillidae ANT 27211 (Current study)	0	0	0	0	0	0	0	1
Cinachyrella alloclada (Uliczka, 1929)	0	0	0	0	0	0	1	0
Cinachyrella paterifera (Wilson, 1925)	0	0	0	0	0	0	1	0
Cinachyrella australiensis (Carter, 1886)	0	0	0	0	0	0	1	0
Cinachyrella levantinensis (Vacelet, Bitar, Carteron, Zibrowius & Perez, 2007)	0	0	0	0	0	0	1	0
Cinachyrella schulzei (Keller, 1891)	0	0	0	0	0	0	1	0
Craniella cranium (Müller, 1776)	0	1	0	0	0	0	0	0
Craniella cf. *leptoderma* (Szitenberg, 2013)	0	0	0	0	0	0	0	1
Craniella sp. QMG 316342 (Szitenberg, 2013)	0	0	1	0	0	0	0	0
Craniella sp. QMG 316372 (Belinky, 2012)	0	0	1	0	0	0	0	0
Craniella sp. ZMBN 85240 (Cardenas, 2011)	0	1	0	0	0	0	0	0
Craniella sp. BIOICE 3659 (Szitenberg, 2010)	0	1	0	0	0	0	0	0
Craniella sp. QMG 318785 (Szitenberg, 2013)	0	1	0	0	0	0	0	0
Craniella zetlandica (Carter, 1872)	0	1	0	0	0	0	0	0
Cinachyrella kuekenthali (Uliczka, 1929)	0	0	0	0	0	0	1	0
Fangophilina sp. (Szitenberg, 2013)	0	0	0	0	0	0	1	0
Geodia cydonium (Jameson, 1811)	0	0	0	1	0	0	0	0
Geodia neptuni (Sollas, 1886)	0	0	0	1	0	0	0	0
Cinachyrella apion (Uliczka, 1929)	0	0	0	0	0	0	1	0
Paratetilla bacca (Selenka, 1867)	0	0	0	0	0	0	1	0
Tetilla leptoderma (Current study)	0	0	0	0	0	0	0	1
Tetilla grandis (Current study)	0	0	0	0	0	0	0	1
Tetilla japonica (Lampe, 1886)	0	0	0	0	0	0	1	0
Tetilla murycii (Peixinho, Pinheiro & Menegola, 2011)	0	0	0	0	0	0	1	0
Tetilla radiata (Selenka, 1879)	0	0	0	0	0	0	1	0
Tetilla sagitta (Cardenas, 2008)	0	0	0	0	0	0	0	1
Amphitetya microsigma (Lendenfeld, 1907)	0	0	0	0	0	1	0	0
Craniella sagitta (Szitenberg, 2013)	0	0	0	0	0	0	0	1

NAME	Corrugated Surface	Smooth surface	Hispid surface	Conulose surface	Hair-like	Short shafted triaenes	Megacanthoxeas	Sigmaspires	Aster	Amphitriaenes
Acanthotetilla celebensis (de Voogd & van Soest, 2007)	0	0	1	0	0	0	1	1	0	0
Acanthotetilla walteri (Peixinho, Fernandez, Oliveira, Caires & Hajdu, 2007)	0	0	1	0	0	0	1	1	0	0
Acanthotetilla seychellensis (Thomas, 1973)	0	0	1	0	0	0	1	1	0	0
Cinachyra antartica (Current study)	0	1	0	0	0	0	0	1	0	0
Cinachyra barbata (Current study)	0	0	1	0	0	0	0	1	0	0
Tetillidae ANT 27211 (Current study)	0	0	1	0	1	0	0	1	0	0
Cinachyrella alloclada (Uliczka, 1929)	0	0	1	0	0	0	0	1	0	0
Cinachyrella paterifera (Wilson, 1925)	0	1	1	0	0	0	0	1	0	0
Cinachyrella australiensis (Carter, 1886)	0	0	1	0	0	0	0	1	0	0
Cinachyrella levantinensis (Vacelet, Bitar, Carteron, Zibrowius & Perez, 2007)	0	0	1	0	0	0	0	1	0	0
Cinachyrella schulzei (Keller, 1891)	0	1	0	0	0	0	0	1	0	0
Craniella cranium (Müller, 1776)	1	0	0	0	0	0	0	1	0	0
Craniella cf. *leptoderma* (Szitenberg, 2013)	1	0	0	0	0	0	0	1	0	0
Craniella sp. QMG 316342 (Szitenberg, 2013)	0	0	1	0	0	0	0	1	0	0
Craniella sp. QMG 316372 (Belinky, 2012)	0	0	1	0	0	0	0	1	0	0
Craniella sp. ZMBN 85240 (Cardenas, 2011)	0	0	1	0	0	0	0	1	0	0
Craniella sp. BIOICE 3659 (Szitenberg, 2010)	0	0	1	1	0	0	0	1	0	0
Craniella sp. QMG 318785 (Szitenberg, 2013)	1	0	0	0	0	0	0	1	0	0
Craniella zetlandica (Carter, 1872)	1	0	0	0	0	0	0	1	0	0
Cinachyrella kuekenthali (Uliczka, 1929)	0	0	1	0	0	0	0	1	0	0
Fangophilina sp. (Szitenberg, 2013)	0	0	1	0	0	0	0	1	0	0
Geodia cydonium (Jameson, 1811)	0	0	1	0	0	0	0	0	1	0
Geodia neptuni (Sollas, 1886)	0	0	1	0	0	0	0	0	1	0
Cinachyrella apion (Uliczka, 1929)	0	0	1	0	0	0	0	1	0	0
Paratetilla bacca (Selenka, 1867)	0	0	1	0	0	1	0	1	0	0
Tetilla leptoderma (Current study)	1	0	0	0	0	0	0	1	0	0
Tetilla grandis (Current study)	0	1	0	0	0	0	0	1	0	0
Tetilla japonica (Lampe, 1886)	0	1	0	0	0	0	0	1	0	0
Tetilla murycii (Peixinho, Pinheiro & Menegola, 2011)	0	1	0	0	0	0	0	0	0	0
Tetilla radiata (Selenka, 1879)	0	1	0	0	0	0	0	0	0	0
Tetilla sagitta (Cardenas, 2008)	1	0	0	0	0	0	0	1	0	0
Amphitetya microsigma (Lendenfeld, 1907)	0	1	0	1	0	0	0	1	0	1
Craniella sagitta (Szitenberg, 2013)	0	0	1	0	0	0	0	1	0	0

NAME	ssr1	ssr2	ssr3	ssr4	ssr5	ssr6	ssr7	ssr8	ssr9	ssr10	ssr11	ssr12	ssr13
Acanthotetilla celebensis (de Voogd & van Soest, 2007)	1	0	0	0	0	1	1	0	0	0	0	0	0
Acanthotetilla walteri (Peixinho, Fernandez, Oliveira, Caires & Hajdu, 2007)	1	0	0	0	0	1	1	0	0	0	0	0	0
Acanthotetilla seychellensis (Thomas, 1973)	1	0	0	0	0	1	1	0	0	0	0	0	0
Cinachyra antartica (Current study)	1	1	1	0	0	0	0	0	0	1	0	0	0
Cinachyra barbata (Current study)	1	1	1	0	0	0	0	0	0	1	0	0	0
Tetillidae ANT 27211 (Current study)	1	1	1	0	0	0	0	0	0	1	0	0	0
Cinachyrella alloclada (Uliczka, 1929)	1	0	0	0	0	1	1	0	0	0	0	0	0
Cinachyrella paterifera (Wilson, 1925)	1	0	0	0	0	1	1	0	0	0	0	0	0
Cinachyrella australiensis (Carter, 1886)	1	0	0	0	0	1	1	0	0	0	0	0	0
Cinachyrella levantinensis (Vacelet, Bitar, Carteron, Zibrowius & Perez, 2007)	1	0	0	0	0	1	1	0	0	0	0	0	0
Cinachyrella schulzei (Keller, 1891)	1	0	0	0	0	1	1	0	0	0	0	0	0
Craniella cranium (Müller, 1776)	0	0	0	1	1	1	0	0	0	0	0	0	0
Craniella cf. *leptoderma* (Szitenberg, 2013)	1	1	1	0	0	0	0	0	0	1	0	0	0
Craniella sp. QMG 316342 (Szitenberg, 2013)	1	1	1	0	0	0	0	0	0	0	1	0	0
Craniella sp. QMG 316372 (Belinky, 2012)	1	1	1	0	0	0	0	0	0	0	1	0	0
Craniella sp. ZMBN 85240 (Cardenas, 2011)	0	0	0	1	1	1	0	0	0	0	0	0	0
Craniella sp. BIOICE 3659 (Szitenberg, 2010)	0	0	0	1	1	1	0	0	0	0	0	0	0
Craniella sp. QMG 318785 (Szitenberg, 2013)	0	0	0	1	1	1	0	0	0	0	0	0	0
Craniella zetlandica (Carter, 1872)	0	0	0	1	1	1	0	0	0	0	0	0	0
Cinachyrella kuekenthali (Uliczka, 1929)	1	0	0	0	0	1	1	0	0	0	0	0	0
Fangophilina sp. (Szitenberg, 2013)	1	1	0	1	1	0	0	0	0	0	0	0	0
Geodia cydonium (Jameson, 1811)	1	0	0	0	0	0	0	1	1	0	0	0	0
Geodia neptuni (Sollas, 1886)	1	0	0	0	0	0	0	1	1	0	0	0	0
Cinachyrella apion (Uliczka, 1929)	1	0	0	0	0	1	1	0	0	0	0	0	0
Paratetilla bacca (Selenka, 1867)	1	0	0	0	0	1	1	0	0	0	0	0	0
Tetilla leptoderma (Current study)	1	1	1	0	0	0	0	0	0	1	0	0	0
Tetilla grandis (Current study)	1	1	1	0	0	0	0	0	0	1	0	0	0
Tetilla japonica (Lampe, 1886)	0	0	0	0	0	0	0	0	0	0	0	1	1
Tetilla murycii (Peixinho, Pinheiro & Menegola, 2011)	0	0	0	0	0	0	0	0	0	0	0	1	1
Tetilla radiata (Selenka, 1879)	0	0	0	0	0	0	0	0	0	0	0	1	1
Tetilla sagitta (Cardenas, 2008)	1	1	1	0	0	0	0	0	0	1	0	0	0
Amphitetya microsigma (Lendenfeld, 1907)	?	?	?	?	?	?	?	?	?	?	?	?	?
Craniella sagitta (Szitenberg, 2013)	1	1	1	0	0	0	0	0	0	1	0	0	0
