# Supplementary material for: Phylogenetic Reassessment of Antarctic Tetillidae (Demospongiae, Tetractinellida) Reveals New Genera and Genetic Similarity among Morphologically Distinct Species
Source: PLoS One. 2016 Aug 24;11(8):e0160718. doi: 10.1371/journal.pone.0160718 (PMC4996456; doi:10.1371/journal.pone.0160718)
Supplement: S2 File — Localities, depths, and Genbank accession numbers of the corresponding sequences are also listed. QMG, Queensland Museum, Brisbane, Australia; NIWA, National Institute of Water & Atmospheric Research, New Zealand; SAM, South Australia Museum; ZMBN, Zoological Museum in Bergen, Norway. In bold, specimens re-examined in this study. Specimens with (*) were only seen on pictures. (PDF) [file pone.0160718.s002.pdf]

Phylogenetic reassessment of Antarctic Tetillidae (Demospongiae, Tetractinellida) reveals new genera and genetic similarity among morphologically distinct species

Carella M[1], Agell G[1], Cárdenas[2,3]P, Uriz MJ[1]*

[1]Centre d'Estudis Avançats de Blanes (CEAB-CSIC). Accés Cala St Francesc 14. 17300 Blanes (Girona) Spain

[2] Département Milieux et Peuplements Aquatiques, Muséum National d'Histoire Naturelle, UMR 7208 "BOrEA", Paris, France

[3]Department of Medicinal Chemistry, Division of Pharmacognosy, BioMedical Centre, Husargatan 3, Uppsala University, 751 23 Uppsala, Sweden

* Corresponding author: losune@ceab.csic.es

S2 file. Original and revised identifications of Tetillidae voucher specimens from previous studies (Cárdenas et al., 2011, Szitenberg et al., 2010, 2013) after morphological re-examination.

Original identification	Szitenberg et al., 2013	Voucher	Locality	Depth (m)	COI Genbank accession #	External features	Identification after revision
Cinachyra* cf. *antarctica	***Craniella sagitta***	**NIWA 28929**	**Antarctica, -71.15, 171.17**	**1158-1165**	**JX177863**	**Dense hispidity.**	**Tetillidae sp. 1**
Craniella sagitta microsigma	***Craniella sagitta***	**NIWA 28491**	**New Zealand, Chatham Rise -45.04, 175.48**	**1239-1251**	**JX177915**	**Dense hispidity, oscules not visible.**	**Tetillidae sp. 2**
Craniella sagitta microsigma	*Craniella sagitta*	NIWA 25206	New Zealand, Chatham Rise -42.79, 179.98	925-1024	JX177917	Very tiny piece.	Tetillidae sp. 2
Craniella cf. *leptoderma*	*Craniella* cf. *leptoderma*	NIWA 27816*	New Zealand, Chatham Rise -43.86, 177.65	582-592	_	Single oscule on top.	*Antarctotetilla leptoderma*
Craniella cf. *leptoderma*	*Craniella* cf. *leptoderma*	NIWA 36097*	Antarctica -75.63, 169.85	525-530	JX177866	Multiple oscules on top.	*Antarctotetilla grandis*
Craniella cf. *leptoderma*	*Craniella* cf. *leptoderma*	NIWA 28910*	Antarctica 71.3°S, 170.5°E	312-323	JX177865	Multiple oscules on top.	*Antarctotetilla* cf. *grandis*
Craniella leptoderma	*Craniella* cf. *leptoderma*	NIWA 28524*	New Zealand, Chatham Rise -44.6, 178.4	1230-1241	JX177895	Single oscule on top.	*Antarctotetilla leptoderma*
Craniella leptoderma	*Craniella* cf. *leptoderma*	NIWA 28507*	New Zealand. Chatham Rise -44.4813347, 177.1430054	1230-1235	JX177896	Single oscule on top.	*Antarctotetilla leptoderma*
Craniella leptoderma	*Craniella* cf. *leptoderma*	NIWA 52077*	New Zealand, Chatham Rise -44.1, 174.4	576-578	JX177916	Single oscule on top.	*Antarctotetilla leptoderma*

Craniella leptoderma	*Craniella* cf. *leptoderma*	NIWA 28496*	New Zealand, Chatham Rise<br>-45.0583344, 175.4743347	1238-1258	JX177897	Dissociated spicule mass.	*Antarctotetilla leptoderma*
Cinachyra barbata	*Cinachyra barbata*	NIWA 28877*	Antarctica, Ross Sea<br>-72.1279983521, 172.700668335	496-501	JX177864	Multiple oscules all over surface, cortex.	*Cinachyra* cf. *barbata*
Cinachyra antarctica	*Cinachyra antarctica*	NIWA 28951*	Antarctica<br>-71.7, 171.14	236-240	JX177868		*Cinachyra antarctica*
Cinachyra antarctica	*Cinachyra antarctica*	NIWA 28957*	Antarctica<br>-71.7, 170.94	127-140	JX177867		*Cinachyra antarctica*
Craniella cf. *leptoderma*	*Craniella* cf. *leptoderma*	QMG 315031*	Antarctica, Casey Antarctic Research Base.	shallow	HM032749	Globular hispid, two large oscules, no cortex.	*Antarctotetilla* cf. *sagitta*
***Craniella* sp. 3878**	***Craniella* sp. 3878**	**QMG 316342**	**Norfolk Ridge**	**400-560**	**HM032747**	**Thin cortex with a palisade of oxeas, numerous porocalices.**	***Cinachyra* sp.**
***Craniella* sp. 3878**	***Craniella* sp. 3878**	**QMG 316372**	**Norfolk Ridge**	**400-560**	**HM032748**	**Thin cortex with a palisade of oxeas, numerous porocalices.**	***Cinachyra* sp.**
Craniella sp. 3318	*Craniella* sp. 3318	QMG 318785*	Norfolk Ridge<br>23° 39' 26.16" S,<br>168° 57.184" E	302-325	HM032752	Small spherical, double-layered cortex, embryos.	*Craniella* sp. 3318
Cinachyra antarctica	*Cinachyra antarctica*	QMG 311149*	Antarctica, McMurdo Base, Ross Island	20	JX177914	Hairy, several oscules.	*Cinachyra* sp.
Fangophilina sp.	*Fangophilina* sp.	NIWA 28586	New Zealand.<br>Challenger Plateau<br>-37.48734, 169.4605	1152-1153	JX177918	Small sample, No porocalices.	cf. *Fangophilina* sp.
***Fangophilina* sp.**	***Fangophilina* sp.**	**NIWA**	**New Zealand.**	**1145-1148**	**JX177919**	**Small sample, No porocalices.**	**cf. *Fangophilina* sp.**

		**28601**	**Challenger Plateau** **-37.1685, 167.7272**				
Fangophilina sp.	*Fangophilina* sp.	NIWA 28614	New Zealand. Challenger Plateau -39.9815, 167.6933	1197-1200	–		*Fangophilina* sp.
Fangophilina sp.	*Fangophilina* sp.	NIWA 28617	New Zealand. Challenger Plateau -39.92617, 167.6933	1139-1144	JX177912	Small sample, No porocalices.	cf. *Fangophilina* sp.
Amphitethya* cf. *microsigma	***Amphitethya* cf. *microsigma***	**SAM S1189**	**South Australia, Great Australian Bight, -34.12120, 133.29300**	**100**	**JX177910**		***Amphitethya microsigma***
Cinachyrella schulzei	*Cinachyrella schulzei*	QMG 320143*	Man and Wife Island, Keppel Islands, Australia -23.12027778, 150.9902778	22	HM032746		*Cinachyrella* cf. *tenuiviolacea*
Cinachyrella schulzei	*Cinachyrella schulzei*	QMG 320636*	Mielaniie Patch, Coral Sea, Australia -14.10266685, 144.5715332	16	HM032745		*Cinachyrella* cf. *tenuiviolacea*
Craniella cranium	***Craniella cranium***	**ZMBN 85239**	**Norway, Korsfjord**	**200-400**	**HM592668**		***Craniella* aff. *zetlandica***
***Craniella* sp.**	***Craniella* sp.**	**ZMBN 85240**	**Norway, Korsfjord**	**200-400**	**HM592669**		***Craniella* cf. *cranium***

[1] but 2 bp. difference with NIWA 36097.

Localities, depths, and Genbank accession numbers of the corresponding sequences are also listed. QMG, Queensland Museum, Brisbane, Australia; NIWA, National Institute of Water & Atmospheric Research, New Zealand; SAM, South Australia Museum; ZMBN, Zoological Museum in Bergen, Norway. In bold, specimens re-examined in this study. Specimens with (*) were only seen on pictures.
