# Supplementary material for: Phylogenetic Reassessment of Antarctic Tetillidae (Demospongiae, Tetractinellida) Reveals New Genera and Genetic Similarity among Morphologically Distinct Species
Source: PLoS One. 2016 Aug 24;11(8):e0160718. doi: 10.1371/journal.pone.0160718 (PMC4996456; doi:10.1371/journal.pone.0160718)
Supplement: S3 File — (PDF) [file pone.0160718.s003.pdf]

Phylogenetic reassessment of Antarctic Tetillidae (Demospongiae, Tetractinellida) reveals new genera and genetic similarity among morphologically distinct species

Carella M[1], Agell G[1], Cárdenas[2],[3]P, Uriz MJ[1]*

[1]Centre d'Estudis Avançats de Blanes (CEAB-CSIC). Accés Cala St Francesc 14. 17300 Blanes (Girona) Spain

[2] Département Milieux et Peuplements Aquatiques, Muséum National d'Histoire Naturelle, UMR 7208 "BOrEA", Paris, France

[3]Department of Medicinal Chemistry, Division of Pharmacognosy, BioMedical Centre, Husargatan 3, Uppsala University, 751 23 Uppsala, Sweden

* Corresponding author: iosune@ceab.csic.es

S3 File. The different parts of the predicted secondary structures (V4 region of 18S) are encicrled and numbered.

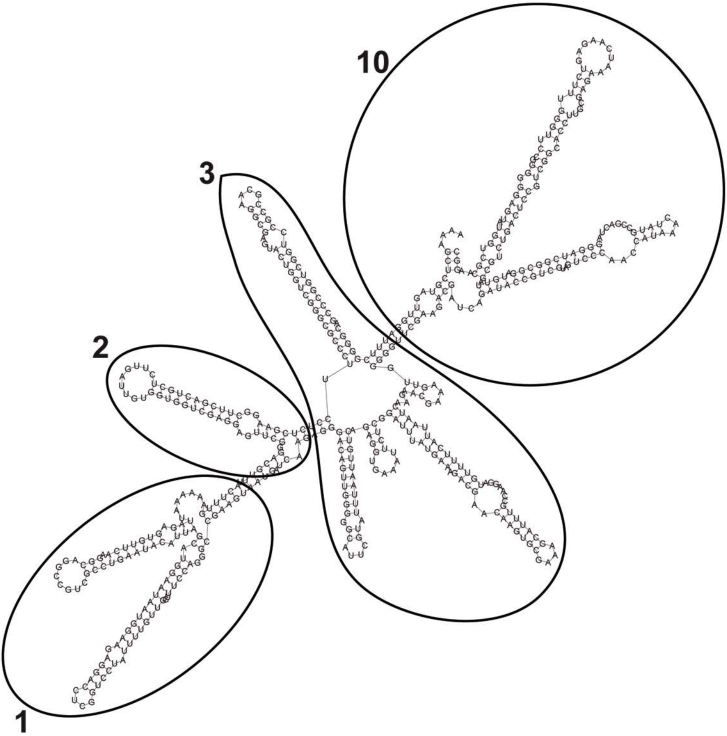

Cinachyra - *Antarctotetilla* - Tetillidae sp.1, sp.2, sp.3.

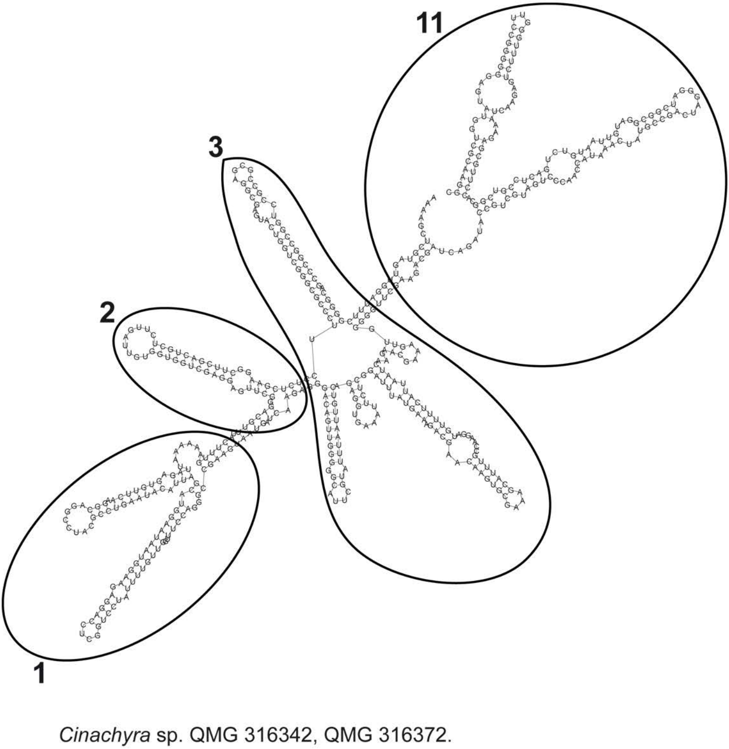

Cinachyra sp. QMG 316342, QMG 316372.

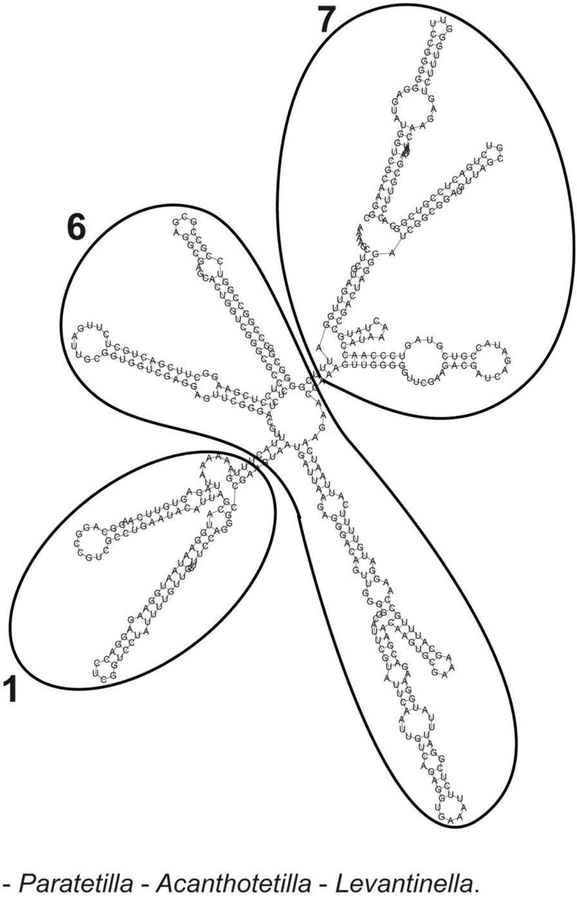

Cinachyrella - Paratetilla - Acanthotetilla - Levantinella.

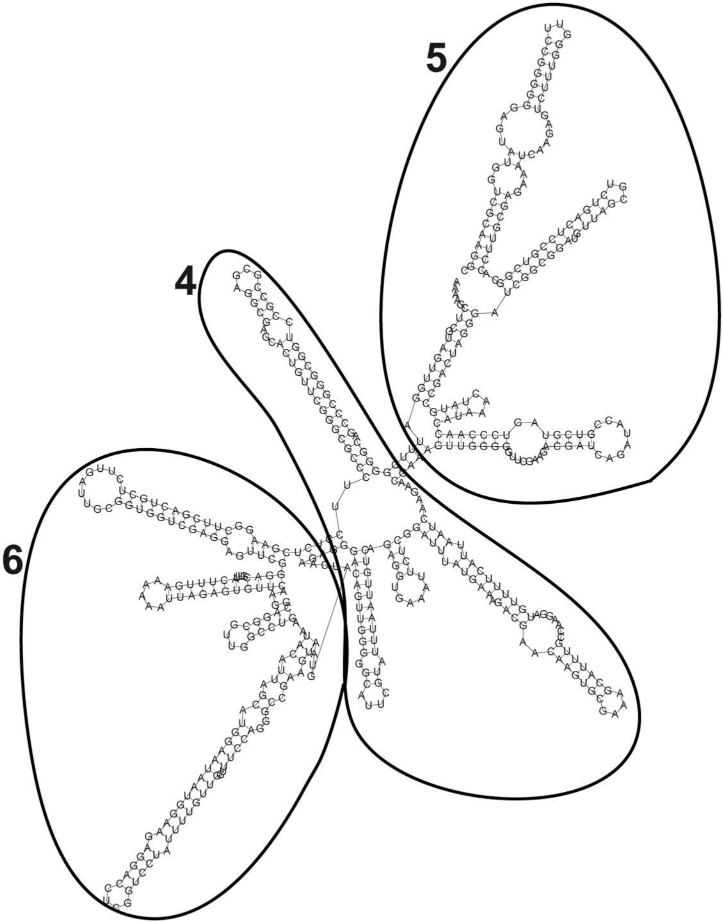

Craniella.

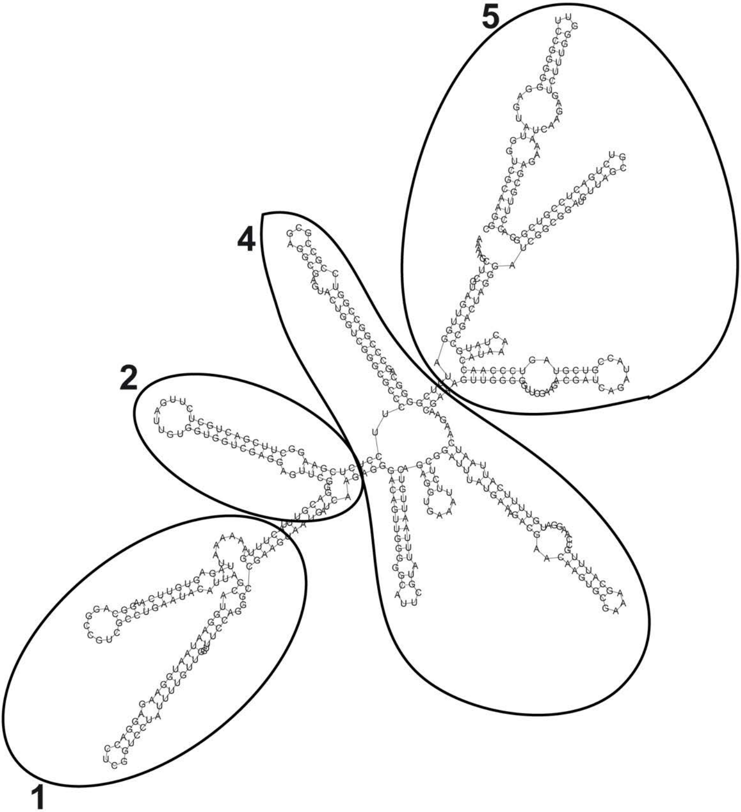

Fangophilina sp.

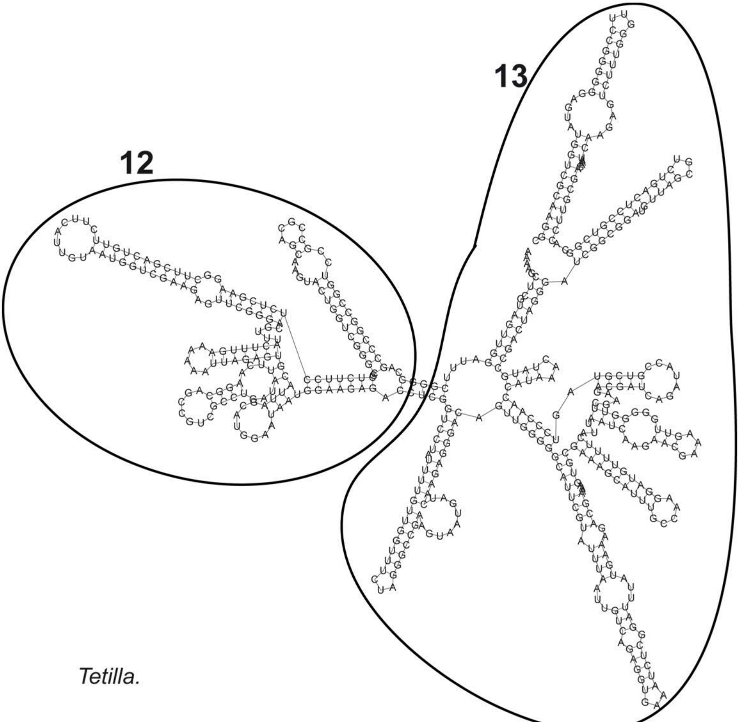

Tetilla.

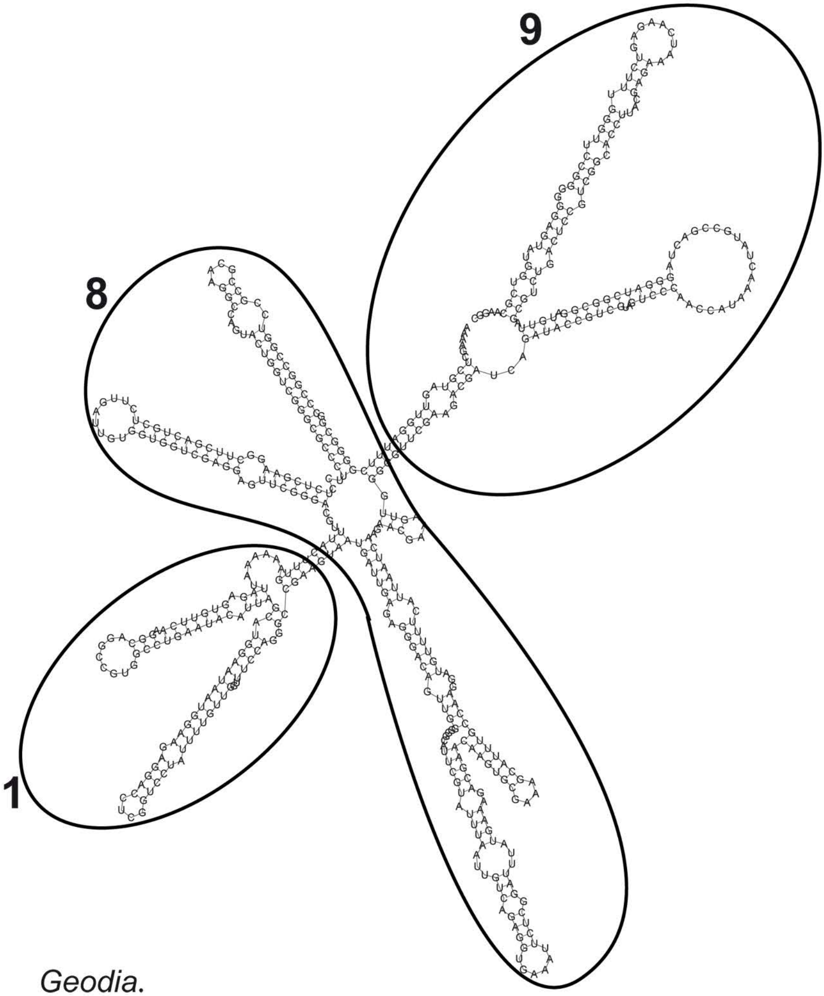

Geodia.
